# Simultaneous free-surface profilometry and subsurface velocimetry with fringe projection and PIV

**DOI:** 10.1007/s00348-026-04242-x

**Published:** 2026-07-28

**Authors:** Ali Semati, Adharsh Shankaran, Benjamin K. Smeltzer, Eirik Æsøy, R. Jason Hearst, Simen Å. Ellingsen

**Affiliations:** 1 Department of Energy and Process Engineering, Norwegian University of Science and Technology, Kolbjørn Hejes vei 2, 7034 Trondheim, Norway; 2 Department of Ships and Ocean Structures, SINTEF Ocean, Professor J.H.L. Vogts veg 1A, 7052 Trondheim, Norway

## Abstract

This work presents a novel combination of two well-established techniques: fringe projection profilometry (FPP) and particle image velocimetry (PIV). Despite seemingly conflicting requirements—FPP requires an opaque surface to project onto, while PIV requires a transparent fluid—both requirements are met by adding low concentrations (4–25 mg/L) of fluorescein dye to the water. This dye strongly absorbs the blue light projected onto the surface for FPP while remaining nearly transparent to the green PIV laser, achieving simultaneous opacity and transparency depending on wavelength. A set of three optical filters suppresses fluorescence-induced noise in the PIV images and specular reflections in the profilometry images, enabling clean simultaneous acquisition. Validated against point laser-induced fluorescence measurements of the surface, the method achieves a mean absolute error in surface elevation of 18 $\upmu \text {m}$ at a dye concentration of 12 mg/L, above which further increases in concentration yield little improvement. PIV correlation values remain robust up to 20 mg/L. The technique resolves surface features differing in amplitude by two orders of magnitude and is demonstrated on flow past a cylinder interacting with surface waves and on droplet impacts on a quiescent surface.

**Supplementary Information:**

The online version contains supplementary material available at https://doi.org/10.1007/s00348-026-04242-x.

## Introduction

Turbulence beneath a free water surface is of high scientific and practical importance. The water-side turbulence controls the exchange of gas and heat between ocean and atmosphere (Zappa et al. 2007; Veron et al. 2011; D’Asaro 2014) and is widely studied numerically (e.g. Kermani et al. 2011; Herlina and Wissink 2019; Pinelli et al. 2022) and experimentally (e.g. Herlina and Jirka 2008; Turney and Banerjee 2013; Bullee et al. 2024; Li et al. 2025; Shankaran and Hearst 2025). The free surface shows distinctive, readily visible imprints of the vortex structure below, and the understanding of the intricate relationship between the two has evolved steadily from primarily qualitative observations to increasingly quantitative studies (Rood 1995; Longuet-Higgins 1996; Brocchini and Peregrine 2001; Banerjee 1994; Babiker et al. 2023; Aarnes et al. 2025), yet there are many more questions than answers. An attractive prospect is remotely observing subsurface turbulence using the free surface as proxy (Muraro et al. 2021), since measurements from above with optical means are fast, inexpensive, and versatile compared to in situ measurements penetrating the surface. Free-surface motions have been used to infer information about bed conditions in open-channel flows, such as bathymetry (Dolcetti et al. 2016; Gakhar et al. 2020, 2022) and submerged canopies (Mandel et al. 2017, 2019).

To understand their interplay, simultaneous high-resolution measurements of the moving free surface and the velocity field underneath are therefore a highly valuable tool for studying the surface–bulk interactions experimentally. In addition to validating numerical work (Shen et al. 1999; Guo and Shen 2010), such experiments also provide physical insight unconstrained by the modelling limitations of simulations. This approach allows one to study not only statistical relationships between surface motion and bulk turbulence, but also the instantaneous relationships between vortices and their signatures. However, such measurements pose a very different set of challenges compared to the more traditional measurements of subsurface turbulence in a vertical plane, where the surface can be traced with fluorescent dye (Smeltzer et al. 2023; Tenhaus et al. 2024).

Techniques for measuring the moving free surface may be broadly grouped into three classes based on their main measurement principle (van Meerkerk et al. 2020; Gomit et al. 2022): stereoscopic, deflection, and projection methods. We briefly review these approaches and their previous combinations with velocity measurements. A fuller overview of surface measurement techniques may be found in the recent survey by Gomit et al. (2022).

Stereoscopic methods track markers of the surface using images from at least one pair of synchronised cameras. A wide variety of markers have been used, ranging from buoyant particles (Douxchamps et al. 2005), surface ripples (Le Page et al. 2024), fluorescent dye (Ihrke et al. 2005), and projected patterns (Tsubaki and Fujita 2005), to temperature differences (Hilsenstein 2005; Savelyev and Fuchs 2018) and even oranges (Bjørnestad et al. 2021). Though they differ in the features they track, these methods invariably use the stereo camera system to triangulate the positions of the markers, resulting in a point cloud to which a surface can be fitted. Simultaneous measurement of surface velocity may be achieved by tracking the features between subsequent frames (Douxchamps et al. 2005; Aubourg et al. 2017; Fujita et al. 2007) while subsurface velocity measurements are rather more involved. Turney et al. (2009) introduced a technique for the simultaneous measurement of surface topography and subsurface velocity for the study of microscale breaking waves. They used a stereo camera setup that imaged fluorescent particles from above the water surface. Refraction of light through the wavy interface distorted the images from the cameras. Cross-correlation of the stereo pair of images resulted in a displacement field that they related to the surface elevation from prior calibration. A second cross-correlation, performed on subsequent temporal frames, provided a velocity field; this was then corrected for refractive distortion using the surface topography calculated in the first step. In this way, the technique draws on principles characteristic of what we refer to as deflection methods.

Ray deflection methods infer free-surface gradients from the refraction or reflection of light at the air–water interface. An early example is the colour-based technique of Zhang and Cox (1994), in which a translucent coloured screen and Fresnel lens, positioned underwater, project collimated coloured beams upwards through the water surface. The colour observed by a camera above uniquely encodes the local surface slope. Although this method is capable of measuring large surface gradients of up to $51^\circ$, rare among deflection techniques, the requirement that only vertical rays reach the camera, in addition to a complex calibration procedure, limits its practical applicability. Dabiri and Gharib (2001) later combined this approach with PIV to correlate the free-surface elevation (obtained by integration) with the subsurface velocity field in a shear layer. A similar combination of deflection measurements and PIV was implemented by Savelsberg et al. (2006), who used a scanning laser beam refracted by the free surface to obtain line measurements of the surface gradient. By invoking Taylor’s frozen turbulence hypothesis, they reconstructed two-dimensional gradient fields from these line measurements.

Among deflection techniques, Background Oriented Schlieren (BOS) (Moisy et al. 2009) is one of the most widely used, due to its simplicity, accuracy, and ease of implementation. In BOS, a reference pattern is placed either above or below the free surface, while a camera observes the pattern from the opposite side, through the interface. For sufficiently small deflection angles, the apparent displacement of the pattern is linearly proportional to the surface gradient, integration of which yields the surface elevation to within an unknown offset. This difficulty in recovering the absolute surface height is a fundamental limitation shared by deflection-based methods, although it can be overcome by anchoring the reconstruction to a single point of known absolute height within the domain.

A notable variant of BOS was demonstrated by Fouras et al. (2008) and later Gomit et al. (2013), who showed that PIV seeding particles can themselves serve as the reference pattern when the laser sheet is oriented parallel to the free surface. A camera below the channel captures standard double-frame PIV images, while a camera above the surface captures a single frame synchronised with one of the two PIV frames. Cross-correlating the simultaneously captured frames from the two cameras yields the surface gradient, obtained at almost no additional cost from an otherwise standard PIV setup, though spatial resolution is limited by the requirement for sufficient seeding density within each interrogation window.

A more severe limitation of deflection methods arises when surface slopes become too large. This causes ray crossing (caustics), which destroys the unique mapping between the pattern and its image and prevents accurate estimation of the displacement. Even in the absence of ray crossing, strong distortions can cause displacement estimation algorithms to fail. As a result, many flows of interest, such as wind-generated surface ripples, are outside the practical range of deflection methods.

Projection-based techniques can accommodate much larger surface gradients. A widely used approach illuminates the free surface with a thin laser sheet to obtain profile measurements. Bonmarin (1989) used this technique for qualitative measurements of breaking waves, and Duncan et al. (1999) added fluorescein dye to the water, which enabled automatic profile detection. This approach, now commonly referred to as laser-induced fluorescence (LIF), is readily combined with PIV when measurements are made in a vertical plane (Buckley and Veron 2017). In this configuration, the same laser can be used for both PIV and LIF, with optical filters preventing cross-contamination between cameras. More recently, van Meerkerk et al. (2020) extended LIF by scanning the laser line in the transverse direction, producing three-dimensional point clouds of the free surface, though with limited resolution in the scanning direction.

Structured light methods analyse the distortion of a pattern projected onto the surface, relative to the same pattern on a flat reference plane. While the projected pattern may take different forms, the underlying principle remains the same: When the incident light is not normal to the surface, a change in surface height induces a lateral shift in the point where the ray intersects the surface, which is recorded by a camera and analysed algorithmically. Various patterns have been employed, including dots, lines, grids and sinusoidal fringes, with a trade-off between robustness and spatial resolution. Simpler patterns, such as dot grids, are computationally efficient and robust but limited in resolution by the dot spacing, whereas sinusoidal fringe patterns provide per-pixel height measurements at the cost of increased computational complexity and sensitivity to large gradients.

The development of Fourier transform profilometry by Takeda et al. (1982) enabled efficient automatic analysis of fringe distortions and has since been applied to a wide range of free-surface flows, including vortex-induced surface depressions (Zhang and Su 2002), dam-break flows (Cochard and Ancey 2008), surface waves (Cobelli et al. 2009), wave turbulence (Herbert et al. 2010; Cobelli et al. 2011), and liquid sprays (Roth et al. 2020).

A key challenge for structured light techniques is the optical transparency of water, which is commonly addressed by adding titanium dioxide particles to render the fluid opaque. However, titanium dioxide can significantly alter surface tension (Przadka et al. 2011) and hinders all optical access to the interior of the fluid. Alternative approaches have also been explored; for example, Roth et al. (2020) employed high concentrations of fluorescein in their experiments (10 g/L, about a thousand times higher than in the present work).

To date, structured light projection methods have not been combined with PIV, primarily because the former require an optically opaque surface, whereas the latter requires transparency. In the present study, we bridge this gap with low concentrations of fluorescein dye (4 to 25 mg/L) and validate the method with independent LIF measurements. The remainder of this paper is structured as follows: Sect. 2 reviews the fundamentals of Fourier transform profilometry and discusses the optical properties of fluorescein dye. The experimental setup and calibration procedure are described in Sect. 3, followed by an error analysis for varying dye concentrations in Sect. 4.1. After measuring the attenuation length of light in the dye solution in Sect. 4.2, we apply the method to wave–vortex interactions behind a cylinder and to droplet impacts in Sect. 4.3. Finally, we provide recommendations for implementing the technique and outline some remaining challenges in Sect. 5.

## Principles

### Fringe projection profilometry

Surface topography is measured by projecting a fringe pattern onto the surface and comparing its distortion to that of the pattern on a flat reference plane which defines the vertical origin. When the projected light is not normal to the surface, changes in elevation produce shifts in the fringe pattern, which are recorded by a camera above. Each observed shift is related to the local surface elevation through a pixel-wise calibration procedure. Although the specific features of the pattern do not affect this relationship, they influence the achievable measurement accuracy for a given computational cost. Sinusoidal fringe patterns are especially well suited for demodulation via the Fourier transform method of Takeda and Mutoh (1983). When projected onto a flat reference plane, the intensity profile is expressed as

1 $$\begin{aligned} I_\textrm{ref} = I_0(x,y) + R(x,y)\cos (2 \pi f_0 x + \phi _0), \end{aligned}$$

where $I_0(x,y)$ is the background illumination intensity, *R*(*x*, *y*) accounts for local variations in fringe amplitude, $f_0$ is the frequency of the fringe pattern, and $\phi _0$ is the initial phase offset.

A perturbation in the surface modifies the phase, resulting in the perturbed intensity profile

2 $$\begin{aligned} I = I_0(x,y) + R(x,y)\cos (2 \pi f_0 x + \phi ). \end{aligned}$$

The surface elevation field is encoded in the phase difference $\Delta \phi (x,y) = \phi - \phi _0$, which is recovered by computing the phase of the complex product

3 $$\begin{aligned} \Delta \phi (x,y) = \textrm{Im}\left[ \log \left( \hat{G}_{\textrm{ref}}^* \cdot {\hat{G}}\right) \right] , \end{aligned}$$

where $\hat{G}_{\textrm{ref}}$ and $\hat{G}$ are the complex signals obtained by Fourier transforming the reference and perturbed images, isolating the spectrum around the carrier frequency $f_0$ (e.g. using a Gaussian or rectangular window), and then taking the inverse transform.

The spectrum of the perturbed image exhibits sidebands around the carrier frequency, whose width increases with the maximum surface slope. For accurate demodulation, these sidebands must not overlap with either the DC component or the second harmonic. The latter condition is generally more restrictive, and Takeda and Mutoh (1983) showed that for their specific imaging geometry, it requires

4 $$\begin{aligned} \left| \frac{\partial h}{\partial x} \right| _{\max } < \frac{1}{3} \cdot \left( \frac{L}{D} \right) , \end{aligned}$$

where *h* is surface elevation, *L* is the distance from the projector exit pupil to the reference plane, and *D* is the distance between the projector and camera.

The complex exponential satisfies

$$e^{x + i(2n \pi + y)} = e^x e^{iy}, \quad n \in \mathbb {Z},$$

so all numbers differing by integer multiples of 2πi in their imaginary part map to the same complex value. Consequently, the complex logarithm is multivalued and in practice is restricted to its principal branch with imaginary range $(-\pi , \pi ]$. When the true phase shift exceeds this range, $\Delta \phi (x,y)$ is discontinuous at the branch cut with jumps of $\pm 2\pi$, a condition known as phase wrapping.

The simplest unwrapping method proceeds line by line through the phase field, adding or subtracting multiples of 2π whenever a jump greater than π is encountered, so that adjacent values differ by less than π. While this row-by-row (or column-by-column) approach is computationally efficient, it is sensitive to noise and sharp phase variations. More robust techniques have been developed at the cost of additional computational complexity (Herráez et al. 2002). For our experiments, the high signal-to-noise ratio and smooth surface meant that the simple line-by-line method proved robust.

Unwrapping produces a continuous phase field, but the result remains indeterminate up to an unknown multiple of 2π. To resolve this ambiguity, the phase must be known at a minimum of one point in the domain. This can be addressed by spatially limiting the projected fringe pattern so that it does not fill the entire field of view. The resulting dark margin along one edge, parallel to the fringes, makes the first fringe line clearly identifiable. The integer multiple of 2π required to correct the phase field is then determined by comparing the position of the first fringe in the deformed image to the reference image.

The phase shift can be related to surface elevation by geometric methods, which require careful placement of the projector and camera and measurement of the distances involved (Takeda and Mutoh 1983; Zappa and Busca 2009), or polynomial methods, where images of the fringe pattern projected onto several parallel planes at known heights are used to relate phase shift to surface elevation for each individual pixel. There are advantages and disadvantages associated with each family of methods. The restrictions imposed by geometric techniques on the alignment of the projector and camera can be difficult to achieve with sufficient accuracy. For example, the method of Takeda and Mutoh (1983) requires that the camera entrance pupil and the projector exit pupil be at the same height with respect to the surface, which is not straightforward to achieve in practice, owing partly to the fact that their exact positions are not always readily apparent. However, if the experimental facility and equipment allow placing the projector and camera far enough from the surface, good accuracy can be achieved with minimal effort.

Polynomial methods for surface reconstruction from phase shift, on the other hand, avoid the strict restrictions on placement of camera and projector, but require images of the fringe pattern on several planes at known heights. Further, the calibration is valid only over the area covered by the calibration plate. In this study, we used a second-order polynomial calibration, discussed further in Sect. 3.3. For a review of calibration methods in FPP, see Feng et al. (2021).

The profilometry results presented here were processed using custom MATLAB code (available online). The code implements both Fourier transform and wavelet transform profilometry (Zhong and Weng 2004; Gdeisat et al. 2006) and supports two phase-to-height conversion methods: the equation of Takeda and Mutoh (1983) and the polynomial method described above, for which a calibration module is included. Phase unwrapping uses the 1D line-by-line method by default, switching automatically to the 2D method of Herráez et al. (2002) if unwrapping errors are detected.

### Fluorescent dye

**Fig. 1:**
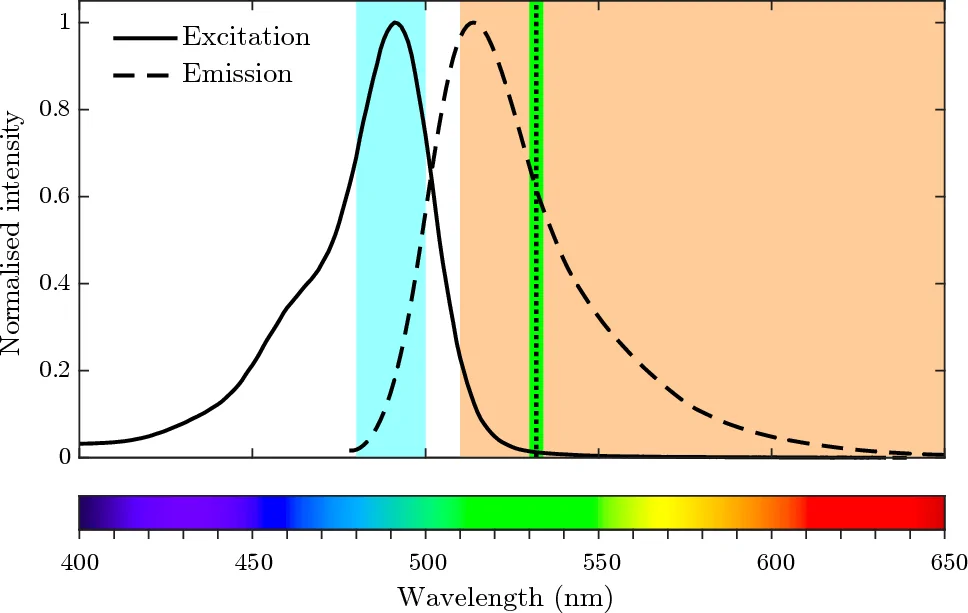
Excitation and emission spectra of fluorescein overlaid with the spectra of the bandpass filters used in this study. Light from the projector is filtered with a 20-nm-wide filter at 490 nm (cyan). A long-pass filter at 510 nm (orange) is placed on the profilometry camera lens, and fluorescent noise is reduced with a 4-nm-wide bandpass filter (green) centred at 532 nm on the PIV camera

The primary challenge of combining projection-based surface measurement with PIV lies in their seemingly conflicting requirements: Surface measurement requires an optically opaque projection surface, whereas PIV requires a transparent medium. These requirements can be reconciled by adding a fluorescent dye to the water that absorbs the projected pattern light while remaining transparent to the laser light.

In most facilities, including ours, PIV is performed using Nd-doped lasers (such as Nd:YAG or Nd:YLF) emitting green light at wavelengths between 527 nm and 532 nm. Unlike LED-based sources, these lasers provide the high power and short pulse duration (on the order of nanoseconds) required for high-accuracy PIV. We therefore restrict our choice of dye to those that interact minimally with green light at 532 nm. The ideal dye has the following properties:

1. Excitation and emission spectra do not overlap with laser light at 532 nm.
2. High quantum yield.
3. Non-toxic and not harmful to the environment.
4. Readily available and cost-effective for larger facilities.

Although a wide variety of fluorescent dyes with diverse optical properties are available, most of these are produced for use in the biomedical sciences and are typically supplied in amounts on the order of milligrammes. In contrast, large-scale hydrodynamic facilities have volumes on the order of 10–100 tonnes, or more requiring hundreds of grammes of dye. Condition #4 thus limits the choice of dye, to our knowledge, to the rhodamine family or to fluorescein. Rhodamine dyes strongly absorb green light and would completely block the 532 nm laser light, leaving fluorescein as the only viable option.

Fluorescein is comparatively inexpensive, produced in large quantities, biodegradable, non-toxic (allowing easy handling and convenient disposal) (Hara et al. 1998), and has a high quantum yield (95%). Its absorption and emission spectra, shown in Fig. 1, are, however, not ideal: Fluorescein absorbs light at 532 nm—weakly, but not insignificantly—and emits primarily in the green, 500 to 550 nm range. This introduces several experimental challenges. Absorption of laser light by the dye reduces the signal-to-noise ratio (SNR) of the PIV images and limits the maximum usable dye concentration. At low dye concentrations, however, the light projected from above penetrates deeper into the water, reducing the contrast of the profilometry images and degrading the accuracy of surface reconstruction with FPP. A balance must therefore be struck between the requirements of the two techniques. Furthermore, excitation of the dye by the laser results in green fluorescence, which appears as noise in the PIV images, further reducing the SNR. We address these challenges through the addition of optical filters to the projector and both cameras, as detailed in Sect. 3.1.

### FPP and semi-transparency

When the water surface is not opaque, light is absorbed and emitted by a subsurface volume of water instead of just the surface layer. Drawing on studies of the error caused by translucency in solids (Lutzke et al. 2011; Xu et al. 2019), we recognise two types of error introduced by semi-transparency. Both share the same physical origin, that is, light interacting with a subsurface volume rather than the surface alone, but manifest differently in the reconstructed surface.

First, subsurface emissions reduce the contrast of the projected pattern. As light emission originates from a subsurface volume rather than the surface alone, a point source projected vertically down onto the surface appears blurred when viewed from above. In the same way, a sine-wave pattern projected onto the surface loses contrast. This loss of contrast makes the measurement system more sensitive to random noise (Xu et al. 2019), since a fixed level of random image noise becomes more significant when the contrast is reduced. Taken to the extreme, the sine wave becomes a flat line and all phase information is lost.

Second, any light ray entering the water excites a column of dye whose centroid of emission lies beneath the surface entry point. This displacement is seen as a false phase shift by the camera whenever its viewpoint differs from that of the projector. The magnitude of the resulting phase error depends on the viewing angle, as shown by Lutzke et al. (2011), the shape of the surface (due to refraction) and, in our case, the dye concentration.

Both effects are governed by how far light travels into the water column before being attenuated, with the former being a limit on precision and the latter being a limit on accuracy. We analyse the effect of dye concentration on reconstruction error in Sect. 4.1 and measure the attenuation length in Sect. 4.2.

## Experimental Setup

**Fig. 2:**
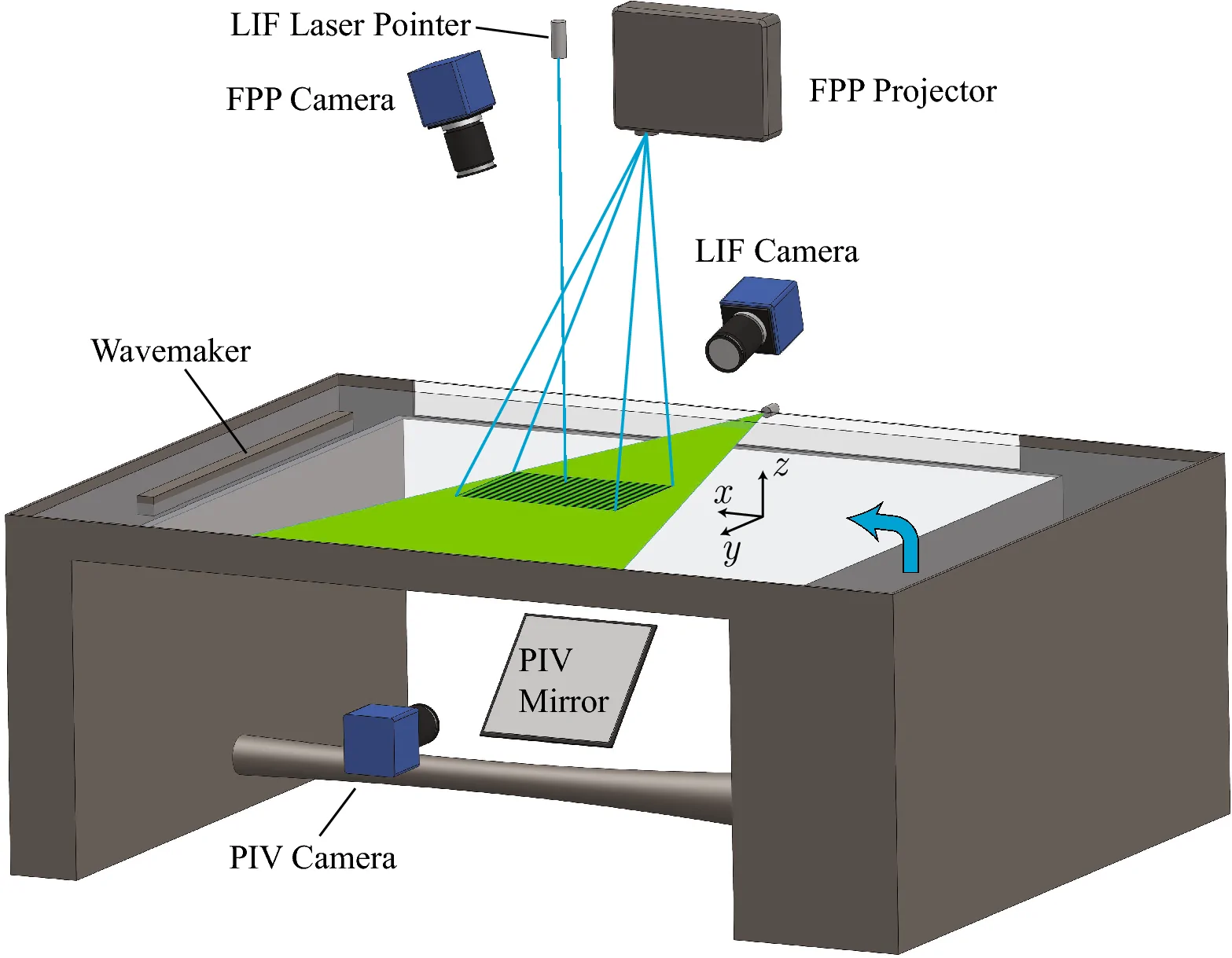
Schematic of the experimental setup. Bandpass filters centred at 532 and 490 nm are placed on the PIV camera and projector, respectively, and a long-pass filter at 510 nm is placed on the FPP camera. The laser pointer and LIF camera were included only in the validation experiments

To validate and demonstrate the measurement technique, experiments were conducted in the small recirculating water channel facility at NTNU. This facility has a $2\times 2\times$ 0.13-m test section with optical access through the base and one sidewall and is equipped with a paddle-type wave generator. A schematic of the setup is shown in Fig. 2, and further details about the flume may be found in Smeltzer et al. (2019a, 2019b).

Fluorescein disodium salt (Thermo Fisher Scientific) was dissolved in the water at concentrations between 4 and 25 mg/L. All imaging was performed using LaVision CX2-25MP cameras. Illumination for PIV was provided by a double-pulsed Nd:YAG laser (Litron Nano L) with a pulse energy of 200 mJ at 532 nm, while sinusoidal stripe patterns (hereafter referred to as “fringes”) were generated by a video projector (Epson EH-TW6700) with a native resolution of 1920×1080 pixels. This projector uses a high-pressure mercury vapour lamp as its light source. Although illumination at 490 nm would be ideal, corresponding to the peak absorption wavelength of fluorescein, the projector spectrum provides sufficient intensity near this wavelength. Mercury vapour lamps have spectral peaks at 403, 435, 546, and 578 nm, but exhibit considerable spectral broadening at higher pressures (Derra et al. 2005), enabling effective excitation of the dye.

Two sets of experiments were conducted: the first to assess the effect of dye concentration on the accuracy of surface measurements and the second to assess its effect on PIV measurements.

To validate the FPP measurements, an independent measurement of surface elevation was obtained by projecting a single beam from a 488 nm continuous-wave laser onto the surface. A camera fitted with a 200 mm lens (the LIF camera in Fig. 2) recorded the laser spot. The high magnification allowed us to extract the surface profile across the spot width (approximately 1.5 mm), from which the local surface elevation and slope were recovered by image analysis. This method is robust to variations in dye concentration and is detailed in Sect. 3.2. For each concentration, the wavemaker was operated at 1 Hz and surface waves were measured simultaneously by both systems at 40 Hz for 20 s. Each measurement was performed twice to ensure repeatability. The exposure times were set to 5 ms for the FPP camera and 2 ms for the LIF camera.

The simplest configuration for simultaneous measurements aligns the PIV and profilometry cameras symmetrically with respect to the water surface, with both optical axes normal to it. However, the bandpass filter placed on the PIV camera reflects the emissions of the fluorescein back towards the profilometry camera in this configuration (see Sect. 3.1 for details). This reflection appears as a circular artefact in the profilometry images and, because the light is refracted by the undulating water surface before reaching the camera, is difficult to remove through post-processing.

This artefact can be eliminated by placing the PIV camera outside the viewing angle of the profilometry camera. For planar PIV, the optical axis must be kept normal to the laser sheet to minimise out-of-plane errors. We thus maintained the PIV camera in its normal orientation and tilted the profilometry camera by $12.5^\circ$ to exclude the PIV camera from its field of view. The profilometry camera was placed 1 m above the water surface. The minimum required tilt angle decreases with increasing camera separation, making it preferable to position the PIV camera as far as possible from the profilometry camera and to use a longer focal length lens to maintain the same field of view. Due to space constraints below the channel, the optical path length between the PIV camera and the laser sheet was extended to 1.7 m using a mirror, as shown in Fig. 2. The PIV and profilometry cameras were equipped with 100 mm and 60 mm lenses, respectively. Note that this geometric constraint is specific to planar PIV in horizontal plane and does not apply to PIV in vertical planes or stereo PIV setups, where the profilometry camera can be oriented normal to the water surface.

The PIV and FPP cameras had fields of view of $207 \times 235\, \textrm{mm}$ and $213 \times 234\, \textrm{mm}$, respectively. The overlap between the two measurement domains was $175 \times 220\, \textrm{mm}$, where the reduced extent in *x* results from an unilluminated margin in the FPP images (see Sect. 2.1).

The effect of fluorescein dye on PIV measurements was assessed by acquiring 500 independent PIV snapshots of uniform flow in a horizontal plane 2 cm below the water surface for each dye concentration. Polystyrene particles with a mean diameter of 40 ${\upmu }\text {m}$ were used as tracers. After applying a min–max pre-processing filter (Adrian and Westerweel 2011), the images were processed using LaVision DaVis 11.0 with a 48×48 pixel (2.1 $\times \ 2.1$ mm) window and 50% overlap. The correlation coefficients were spatially and temporally averaged to yield a single metric representing PIV quality.

### Optical filters

We here detail how a succession of challenges are overcome using three optical filters: an ultranarrow bandpass filter on the PIV camera, a bandpass filter on the projector, and a long-pass filter on the profilometry camera (cameras are indicated in Fig. 2).

**Fig. 3:**
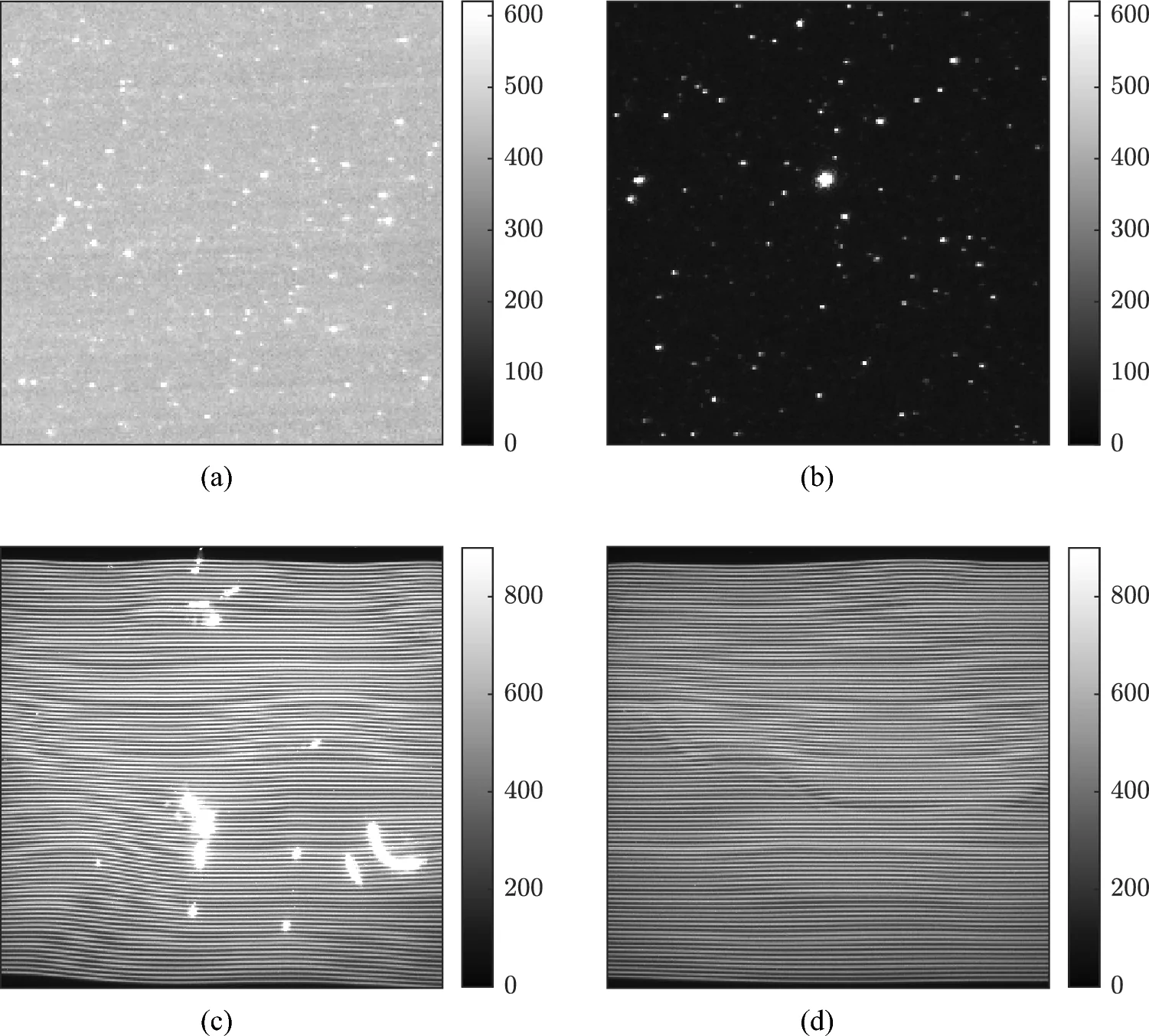
Optical challenges associated with this experimental approach. **a** PIV image showing noise which stems from the absorption of laser light at 532 nm by fluorescein. **b** PIV image taken under the same conditions as (a), but with an ultranarrow bandpass filter with a full-width half-maximum of 4 nm which removes most of the noise. **c** Profilometry image of the fringe pattern shows specular reflections from the water surface which saturate parts of the camera sensor. **d** Profilometry image with the addition of a long-pass filter at 510 nm to the camera, eliminating the specular reflections

The spectral properties of fluorescein dye present challenges for both PIV and profilometry. Figure 3a shows a PIV snapshot acquired without additional filtering. Because fluorescein has small but non-zero absorption at 532 nm as Fig. 1 shows, excitation of the dye by the intense laser light appears as background noise, reducing the signal-to-noise ratio (SNR). Both the laser light and the fluorescence are green; hence, standard PIV bandpass filters are ineffective. However, we can exploit the difference in spectral width between the two sources: The laser light is narrowband (532±0.25 nm), whereas the dye emission has a comparatively broad spectrum spanning the whole green range and beyond (see Fig. 1), only a tiny fraction of which lies within the laser’s spectral band. We therefore employ a 4-nm-wide bandpass filter (RET532/4x, Chroma Technology) centred at 532 nm for PIV. As shown in Fig. 3b, this filter eliminates nearly all of the background noise.

**Fig. 4:**
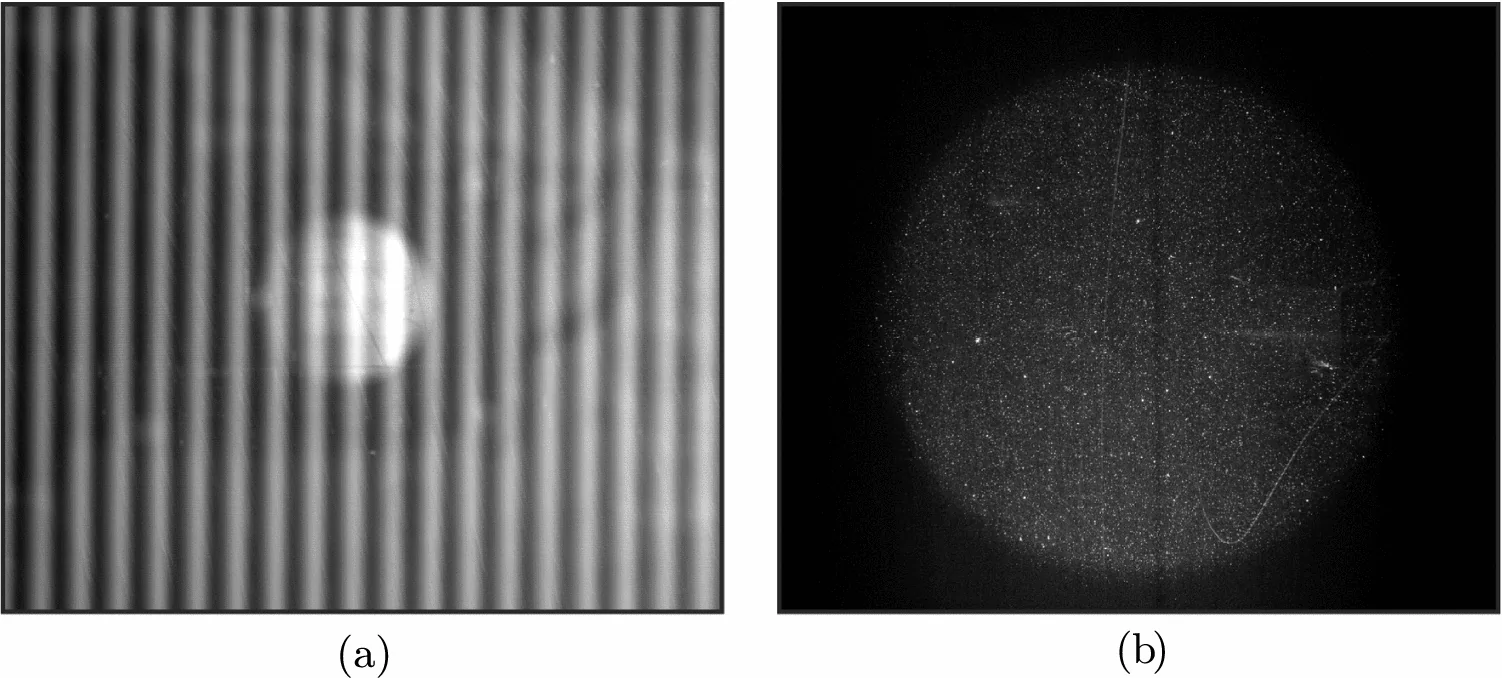
**a** Profilometry image taken from above the water surface showing reflection of light from the ultranarrow bandpass filter on the PIV camera below the surface. **b** PIV image showing cut-off of light when using a wide-angle lens with the ultranarrow filter

The narrowband filter also prevents contamination of the PIV images by the profilometry fringe pattern, which would otherwise necessitate strobing the projector in sync with PIV acquisition or using a mechanical shutter. During the short exposure time of the first frame in double-frame imaging (typically microseconds), the contribution of the projector is negligible compared to the high-intensity Nd:YAG laser pulse (nanosecond duration). However, the second frame presents a specific challenge. The exposure time of the second frame is dictated by the sensor readout time, approximately 100 ms for our cameras. Over this longer duration, the integrated intensity of the continuous projector light becomes significant, making the fringe pattern visible. By blocking most of the fluorescence, the narrowband filter makes the pattern invisible to the PIV camera, allowing for continuous projection and simplifying the experimental setup.

Using an ultranarrow filter does introduce two limitations. First, as noted in Sect. 3, the filter reflects fluorescence and appears as a bright artefact to the profilometry camera unless the setup avoids mirror symmetry. Figure 4a shows a profilometry image acquired with the cameras in mirror opposition, illustrating this reflection. Second, the spectral properties of the filter depend on the angle of incidence. Manufacturers typically report performance for orthogonal incidence; at oblique angles, the passband shifts, potentially blocking the monochromatic laser light. This may be encountered when using wide-angle lenses, as shown in Fig. 4b, where an image taken with a 28 mm focal length lens exhibits a vignetting effect. The darkening on the edges is due to spectral cut-off, not physical obstruction. For PIV photography, the filter thus limits the maximum angular field of view that can be achieved.

As discussed in Sect. 2.2, increasing the concentration of dye makes the surface more opaque to the projected fringe pattern, improving surface reconstruction with FPP. However, absorption of laser light by fluorescein places an upper limit on dye concentration. This limit depends on laser power, PIV particle size (larger particles scatter more light), and the path length of laser light in water (from the laser to the imaging region and from the particles to the camera). Placing a narrow bandpass filter on the PIV camera as described earlier extends the maximum dye concentration with which PIV measurements of acceptable quality can be obtained. Similarly, the minimum dye concentration for reliable profilometry can be lowered by placing a bandpass filter on the projector centred at the maximum absorption wavelength of fluorescein (≈490 nm, Fig. 1). At the same dye concentration, restricting the spectral composition of the projected light to those wavelengths most strongly absorbed by fluorescein will result in a lower penetration depth for the light and thus increase the contrast. We selected a bandpass filter (MV490/20, Chroma Technology) centred at 490 nm with a full-width at half-maximum (FWHM) of 20 nm.

The final challenge arises from specular reflections from the water surface. These reflections, shown in Fig. 3c, can saturate the image sensor and prevent reconstruction of affected regions. Specular reflections are a common obstacle in FPP measurements of polished or metallic surfaces, and various mitigation strategies have been proposed in the literature (Nayar and Gupta 2012; Song et al. 2017). A common approach is to linearly polarise the projected light (Salahieh et al. 2014; Dave et al. 2022). Since specular reflections largely retain the incident polarization, they can be filtered by a cross-oriented polariser on the camera. As the dye emits unpolarised light, this approach is applicable to our system but comes with a severe penalty: The combination of polarisers attenuates the fluorescence signal by a theoretical minimum of 75%.

We therefore adopt an alternative approach (Roth et al. 2020), exploiting the Stokes shift of fluorescein. Because the emission peak occurs at a longer wavelength than the absorption peak, a long-pass filter (ET510lp, Chroma Technology) at 510 nm transmits the majority of emitted light while blocking the reflected excitation light. We observed a 23% reduction in signal intensity after placing the long-pass filter on the profilometry camera, consistent with the 25% reduction predicted by numerically integrating the emission spectrum above 510 nm. This long-pass filter does not, however, block the PIV laser light, and therefore, the FPP camera’s exposure should not overlap with either laser pulse. This can be achieved by synchronising the FPP camera so that exposure occurs before or after the two laser pulses, or between them if the exposure time is sufficiently short.

The optical density (OD) of both the profilometry and projection filters is critical in blocking specular reflections. We found that the OD3 rating of the MV490 filter mentioned above was insufficient to prevent out-of-band transmission from the projector, which appeared as specular reflections. The ET480/40x, however, centred at 480 nm with a FWHM of 40 nm and rated OD6, combined with the camera’s OD6 long-pass filter, completely eliminated the specular reflections, as shown in Fig. 3d. To achieve both the necessary spectral narrowness and high blocking efficiency, we stacked the two projector filters. In this configuration, the ET480/40x provided the optical density required to eliminate the specular reflections, while the MV490/20 constrained the bandwidth.

### Point LIF validation

To validate the FPP method, we performed simultaneous, independent point measurements of the free surface using an optical wave gauge (point-LIF). A 488 nm continuous-wave laser pointer from Zeus lasers (the LIF laser pointer in Fig. 2) was positioned above the water surface and oriented vertically downwards to project a spot of light onto the interface, which was imaged at 40 Hz. Although dye concentration affects the penetration depth of light into the water, the point-LIF method relies only on detecting a sharp intensity transition at the interface and is therefore not sensitive to this effect.

**Fig. 5:**
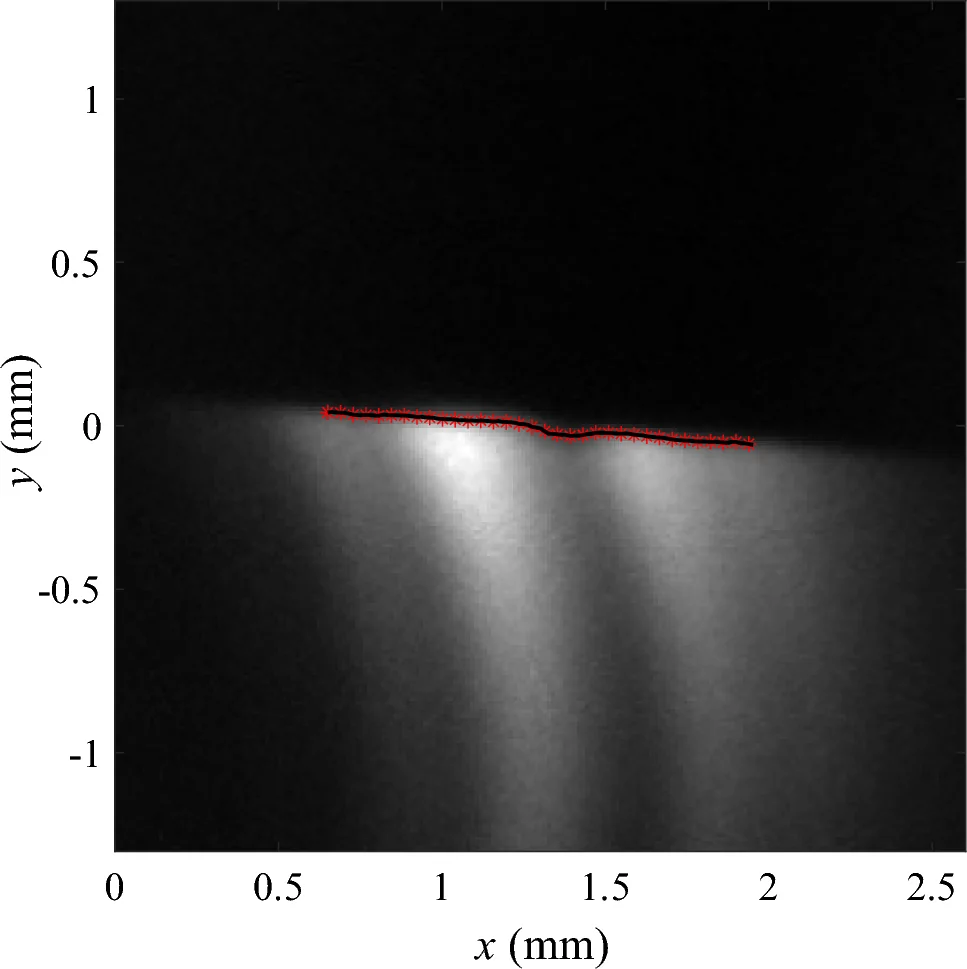
Representative side-view image from the point-LIF camera showing the air–water interface. A vertical laser beam illuminates the water column from above, exciting the fluorescent dye along its path. The detected air–water interface is overlaid as a black line with red markers

A sharp increase in pixel intensity occurs at the air–water interface as the laser beam excites the fluorescent dye, and a simple threshold operation followed by contour detection can be used to identify the interface. However, since the dye concentration varies between experiments, such an approach would require case-specific thresholds. Further, the laser beam was spatially inhomogeneous, likely due to imperfect collimation or lens aberrations, as visible in Fig. 5.

We therefore opted for a gradient-based approach, commonly used for edge detection in computer vision, in which the interface was identified as the location of the maximum vertical intensity gradient. Intensity profiles were extracted along vertical columns of each image and were smoothed using a third-order Savitzky–Golay filter to reduce the noise amplification inherent in numerical differentiation. In Fig. 5, the detected free surface has been overlaid on the image with red markers. The high camera resolution (13 $\upmu \text {m}$ per pixel) provided over 100 points across the 1.5 mm width of the laser spot, sufficient for a robust linear fit to extract both local slope and elevation.

The high-intensity laser spot was also visible in the FPP images, preventing direct comparison at the exact measurement point. We therefore sampled the FPP data at points 4 mm on either side of the laser spot and interpolated the value at the centre. This approach proved robust and accurate, with significant deviations observed only towards the end of each run, when wave reflections from the sidewalls degraded the interpolation accuracy (discussed further in Sect. 4.1).

### Calibration

The PIV and FPP cameras were calibrated using a two-level, double-sided calibration plate (LaVision Type 20) mounted on a translation stage. The bottom surface of the plate was first aligned with the laser sheet (60 mm above the channel bed), after which the plate was raised to align the top surface with the quiescent water level (80 mm above the channel bed). A third-order polynomial model was used for the PIV camera, while a pinhole model was applied to the FPP camera. The point-LIF camera was calibrated separately using a smaller two-level plate (LaVision 106-10) and a third-order polynomial model.

The phase–elevation mapping for the profilometry system was established by projecting the fringe pattern onto a flat white plate mounted on a translation stage. Images were recorded at ten vertical positions spanning the quiescent water level of 80 mm, with a step size of 5 mm. These images were first dewarped and then demodulated to produce phase maps for each height. A second-degree polynomial was then fitted to the phase evolution at each pixel, allowing for the recovery of surface elevation from phase data.

We note that, instead of a machined plate, a container with a thin layer of high-concentration dye solution can be used for this calibration. This approach offers two advantages: The liquid surface is self-levelling, and the calibration area can be made as large as required without the manufacturing constraints of a solid plate. A third alternative, simpler than the former two and particularly useful when a translation stage is impractical, utilises the point-LIF system. The water level is raised to the maximum measurement height, and the channel is slowly drained while the FPP and point-LIF cameras record at fixed intervals. This yields images spanning the full measurement range and requires no manual intervention beyond opening the drain valve.

## Results and Illustrative Applications

### Error analysis

**Fig. 6:**
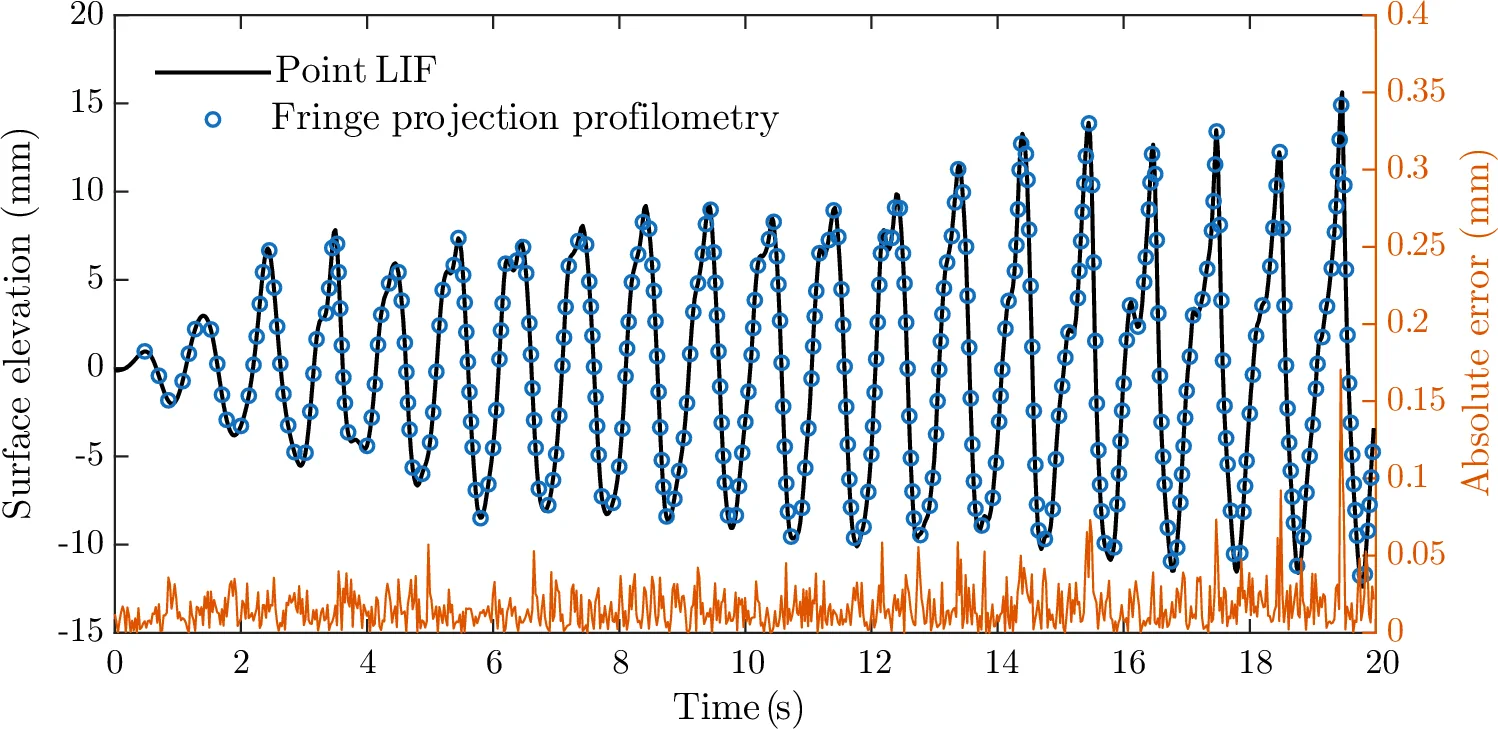
Time series of surface elevation measured by point-LIF and fringe projection profilometry (FPP) at a dye concentration of 12 mg/L. The absolute difference between the two measurements is shown in orange using the right axis (note the smaller scale)

To assess the accuracy of the FPP method, we compared simultaneous measurements by FPP and point-LIF of water waves generated in initially quiescent water by a wavemaker operating at 1 Hz. This procedure ensured that the waveforms were reproducible across runs with different dye concentrations. For each concentration, two sequences of 20-s duration were acquired at a frame rate of 40 Hz.

We calculated the characteristic error using amplitude-based binning. This was to ensure that the error analysis represented the full dynamic range of the wave. The absolute difference between FPP and point-LIF data, the error, was partitioned into eight bins according to surface elevation. The error was averaged within each bin, and the mean of these eight bin-averages was defined as the characteristic error corresponding to a particular dye concentration.

Figure 6 shows a time series of surface elevation measured by the two systems at a dye concentration of 12 mg/L (left axis), with the absolute difference plotted on the right vertical axis. The mean absolute error for this time series is 18 $\upmu \text {m}$, with spikes up to ${\approx 160}\,\upmu \text {m}$ towards the end of the run. As mentioned in Sect. 3.2, the presence of the point-LIF laser spot prevented FPP measurement at the exact same location, necessitating interpolation using data from adjacent points. The observed spikes are the result of interpolation error. While waves propagate essentially unidirectionally along the channel centreline at the start of a run, reflections from the walls eventually create a complex wave field. Sidewall reflections, in particular, induce significant transverse surface undulations, degrading the accuracy of the linear interpolation. We investigated this effect by successively reducing the distance between the two FPP locations and the point-LIF target, calculating the error at each step. This revealed a trade-off: Each reduction decreased the interpolation error but simultaneously increased the mean absolute error due to optical interference from the laser spot in the FPP demodulation process. The 4 mm separation employed in this study was therefore selected as a compromise between these competing sources of error.

**Fig. 7:**
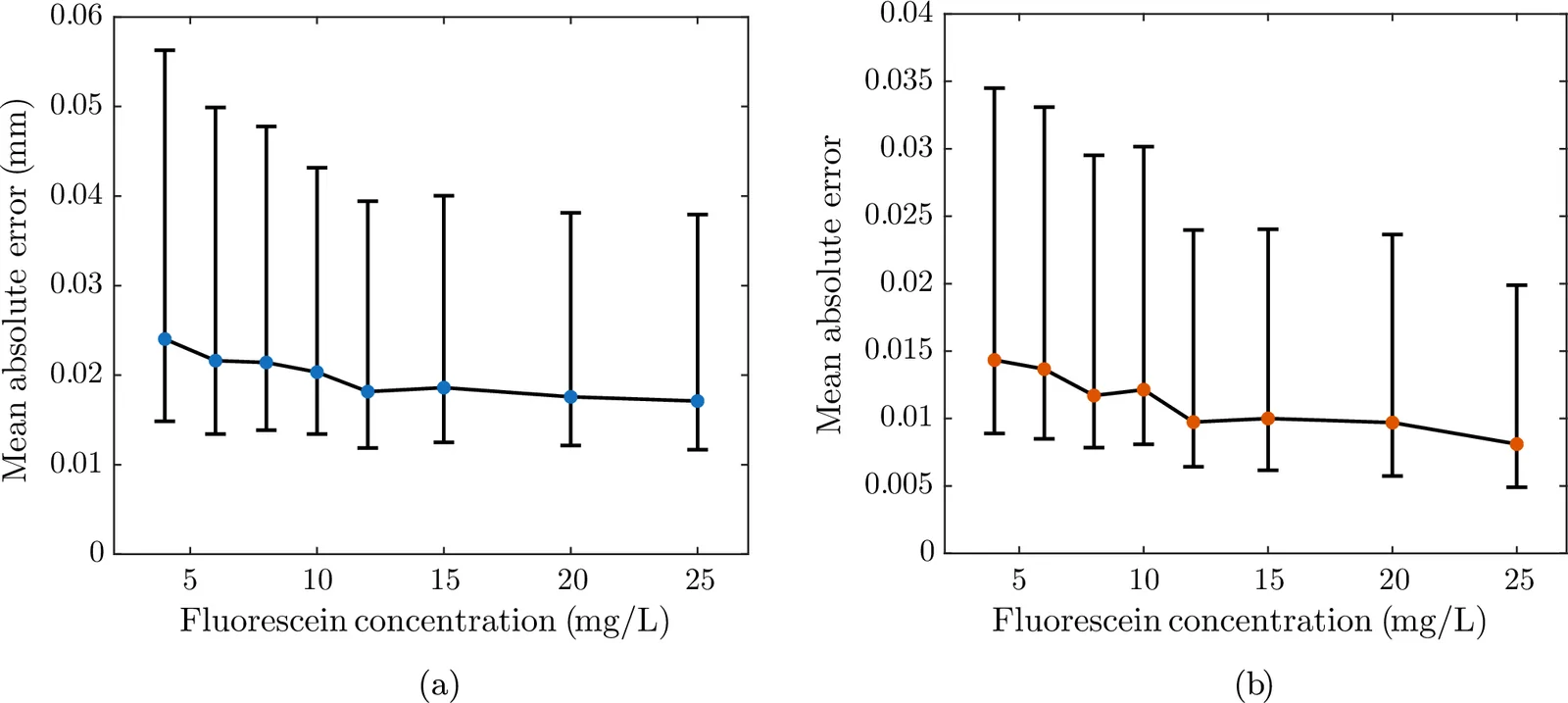
Variation in mean absolute error of **a** surface elevation and **b** slope with dye concentration. In both figures, the interquartile range (25th to 75th percentiles) of the absolute error is indicated by the error bars

Figure 7a shows the variation of mean absolute error (MAE) in surface elevation with dye concentration. The error bars indicate the interquartile range (25th to 75th percentiles). We observe a modest decrease in MAE with increasing dye concentration until it stabilizes at approximately 12 mg/L, beyond which the error remains at a plateau of about 18 $\upmu \text {m}$. The 75th percentile of the absolute error decreases more rapidly than the mean, which indicates suppression of large outliers rather than uniform improvement, though this too stabilizes near 12 mg/L. To interpret this error plateau, we consider the intrinsic uncertainties of the measurement techniques. The noise floor of the FPP system (measured as the standard deviation of a quiescent water surface) is $\sigma _{\textrm{FPP}} = {8}\,\upmu \text {m}$. The uncertainty of the point-LIF system is more difficult to quantify, but can be estimated as ±1 pixel, or 13 $\upmu \text {m}$. Thus, the lower bound for the MAE is approximately 12 $\upmu \text {m}$ (derived from $\sqrt{2/\pi } \sqrt{\sigma _{\textrm{FPP}}^2 + \sigma _{\textrm{LIF}}^2}$), close to the 18 $\upmu \text {m}$ plateau. The remaining gap between the two is likely attributable to residual calibration errors rather than insufficient dye concentration. Notably, even at the lowest concentration, where light penetration is deepest, the 75th percentile of the elevation error remains below 60 $\upmu \text {m}$. Largely similar trends are observed for the MAE of the slope (Fig. 7b), with a modest decrease and a plateau at 12 mg/L.

**Fig. 8:**
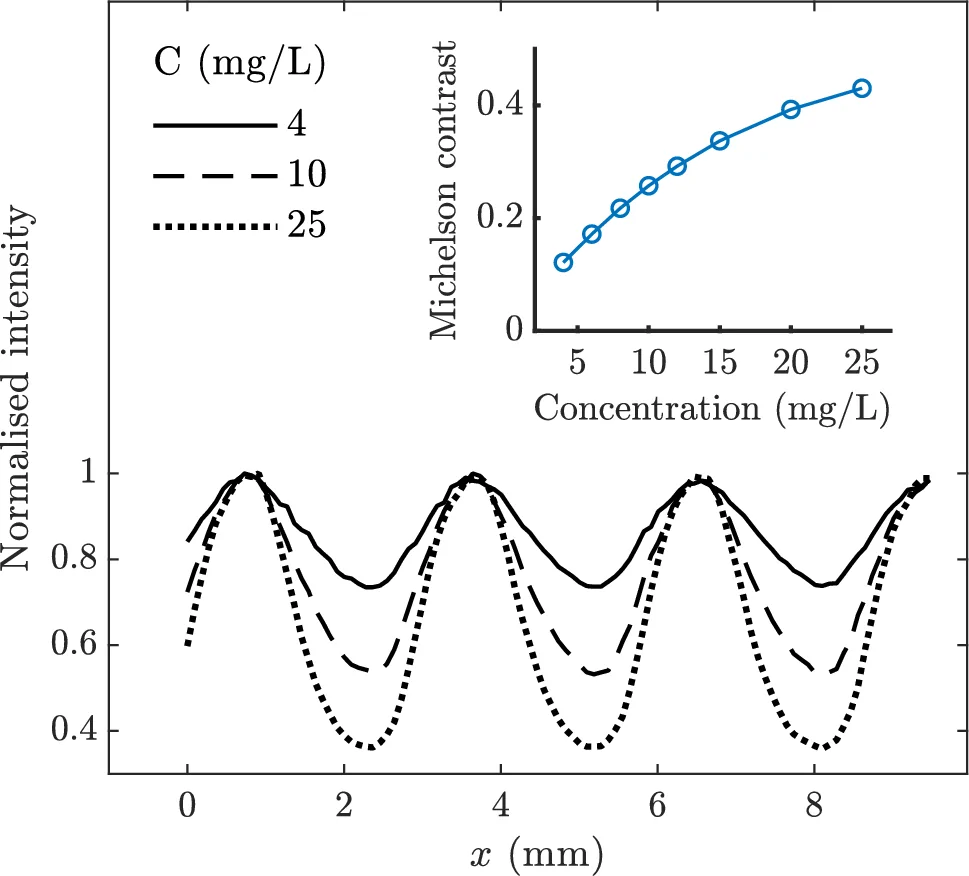
Normalised fringe pattern intensity at three dye concentrations (C = 4, 10, and 25 mg/L). The inset shows the Michelson contrast as a function of concentration

To quantify the contrast of the fringe pattern, we utilised the Michelson contrast, defined as $(I_\textrm{max} - I_\textrm{min})/(I_\textrm{max} + I_\textrm{min})$, where $I_\textrm{max}$ and $I_\textrm{min}$ are the local maximum and minimum intensities. Figure 8 shows the normalised intensity profile for a segment of the fringe pattern at three different concentrations; the inset presents the Michelson contrast as a function of concentration. Despite an almost fourfold increase in contrast from the lowest to the highest concentration, the 75th percentile of the absolute error varies by only 16 $\upmu \text {m}$.

Note, however, that these contrast values were obtained for a quiescent surface. Steep waves or surface roughness can significantly degrade contrast, potentially to the point where the fringe is no longer locally discernible and the demodulation step fails. We therefore recommend a minimum dye concentration of 8 mg/L to ensure robust phase recovery.

The addition of fluorescein dye degrades PIV signal quality through two primary mechanisms: (1) Excitation of the dye by the laser generates background noise, reducing the SNR, and (2) absorption of laser light by the dye attenuates the laser sheet, further reducing particle visibility. As discussed in Sect. 3.1, while the attenuation of laser intensity cannot be avoided, the fluorescence-induced background noise can be mitigated with an ultranarrow bandpass filter.

**Fig. 9:**
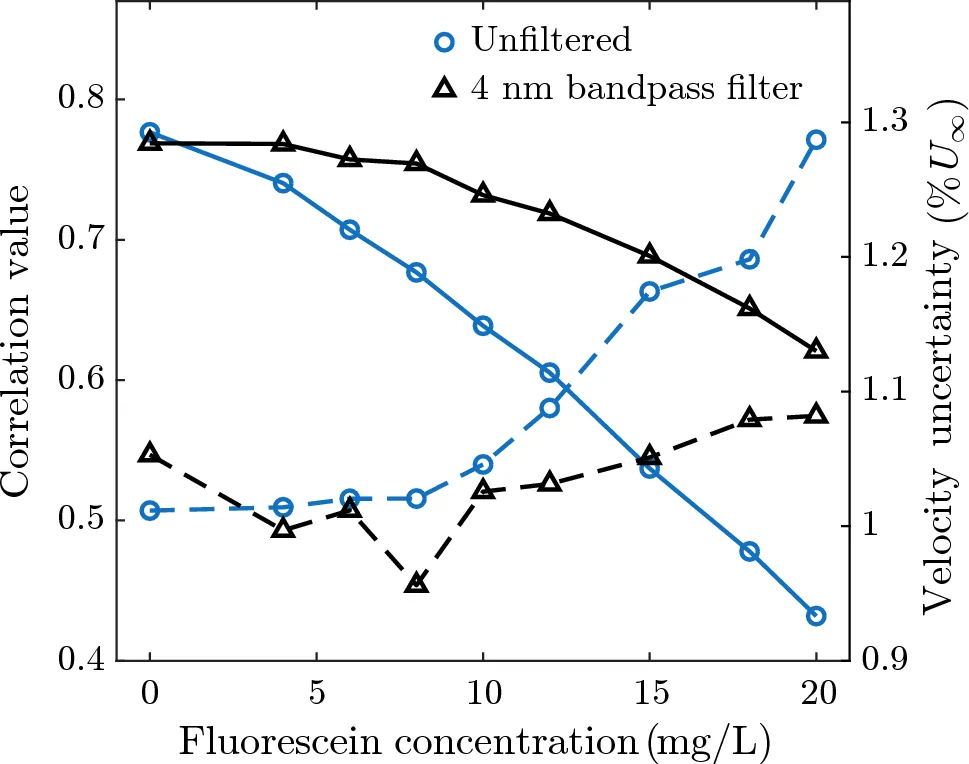
Spatially and temporally averaged PIV correlation values (solid lines, left axis) and uncertainty of the velocity magnitude as a percentage of the free-stream velocity (dashed lines, right axis) for a uniform flow with and without a bandpass filter on the camera

To quantify the efficacy of this filter, 500 image pairs were acquired under uniform flow conditions across a range of dye concentrations, both with and without the bandpass filter, as detailed in Sect. 3. The correlation value was used as a proxy for PIV quality and averaged over time and space to yield a single representative value for each dye concentration, as shown on the left axis in Fig. 9. In addition, the uncertainty of the velocity magnitude, calculated from correlation statistics by DaVis 11.0 and shown by Wieneke (2015) to be a good estimate of measurement error, is displayed on the right axis as a percentage of the free-stream velocity magnitude, likewise averaged in time and space.

Without fluorescein dye, the mean correlation value is approximately 1% higher for the unfiltered configuration. This is expected, as the bandpass filter is not perfectly transmissive, resulting in reduced particle image intensity. Once dye is added, however, the filtered case exhibits higher correlation values, with the gap between the two configurations widening as concentration increases. The decline in correlation observed for the filtered case is almost entirely due to the attenuation of the laser sheet by the dye. In contrast, the more rapid degradation in the unfiltered case, and the resulting performance gap, is driven by the background noise generated by fluorescence.

We note that sufficiently high correlation values can still be obtained without a bandpass filter at lower dye concentrations or for shallow flow depths. Therefore, while an optical bandpass filter consistently improves PIV quality, it may not be strictly necessary to achieve measurements of acceptable accuracy.

A similar divergence between unfiltered and filtered configurations is observed in the velocity uncertainty (dashed lines in Fig. 9). The uncertainty remains nearly constant across the full concentration range with a bandpass filter, but increases by 0.3% without one. While the trend confirms the filter’s effect, the increase in error is negligible in this setup, which we attribute to idealized experimental conditions, namely a shallow optical path (6 cm of water), large seed particles (40$\upmu \text {m}$) combined with high laser power, and uniform flow. Under conditions of greater depth, smaller particles, or higher dye concentration, the benefit of the bandpass filter would be considerably larger.

### Attenuation length

Subsurface emission of light from the dye reduces the accuracy of surface reconstruction (Sect. 2.3). We quantify the subsurface emissions using the attenuation length ℓ, the distance over which beam intensity falls to 1/*e* of its value at the surface. Absorption follows the Beer–Lambert law, $\log _{10}(I_0/I) = \varepsilon c d$, where $I_0$ and *I* are light intensities at entry and after path length *d*, *c* is the molar concentration, and ε is the molar extinction coefficient. The attenuation length defined here is related to the molar extinction coefficient ε by $\ell = (\ln (10)\,\varepsilon c)^{-1}$. The molar concentration was calculated using a molar mass of ${376.2}\,\text {g mol}^{-1}$.

**Fig. 10:**
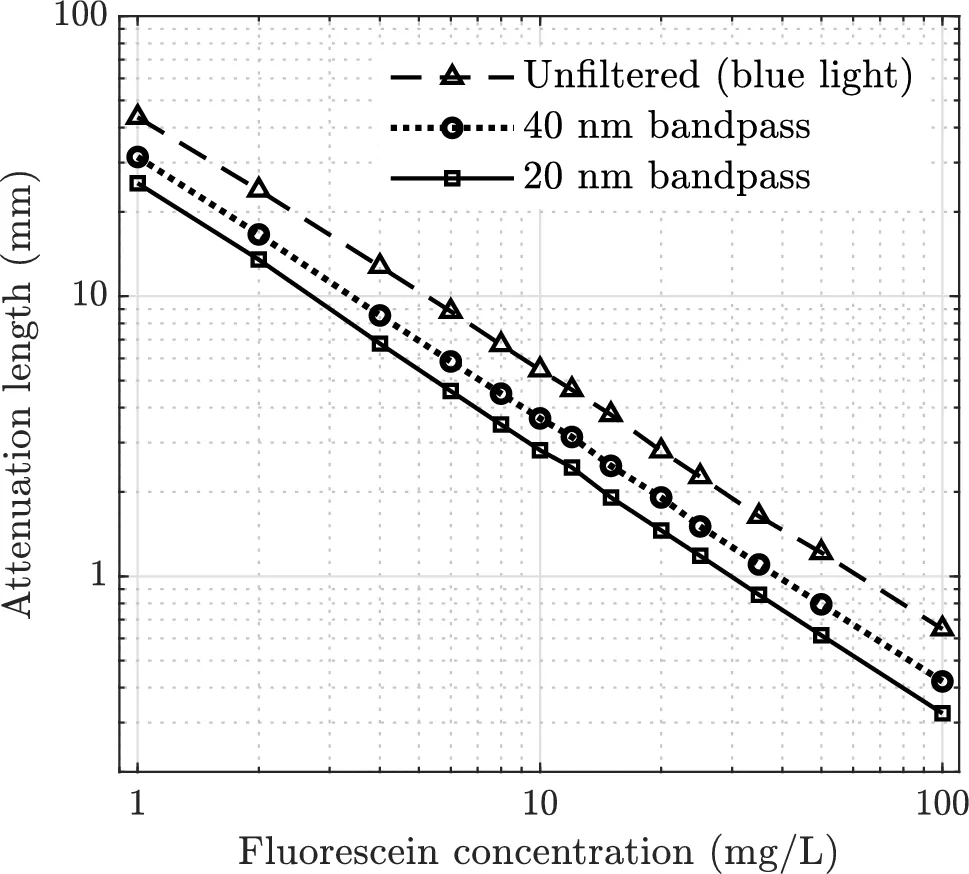
Attenuation length as a function of dye concentration for three projector filter configurations

Since the molar extinction coefficient is tabulated for monochromatic excitation at the peak absorption wavelength and is highly pH sensitive for fluorescein (Mota et al. 1991), we measured the attenuation length directly. A beam from the projector was directed through the left wall of a glass tank of dye solution, propagating horizontally, parallel to and 1 cm away from the front wall, through which a camera (LaVision sCMOS CLHS) imaged the beam path. We tested three filter configurations: unfiltered, 40 nm bandpass, and 20 nm bandpass (see Sect. 3.1 for details on filters), with results shown in Fig. 10. The spectral composition of the blue light for the unfiltered case depends on the projector’s light source and the dichroic mirrors used for colour separation, details of which were not available from the manufacturer, but should be representative of typical lamp-type commercial projectors.

Relative to the unfiltered configuration, the 40  nm and 20  nm bandpass filters reduced the attenuation length by 33% and 47%. A linear fit to $\log _{10}{(I_0/I)}$ vs. *d* yielded *effective* molar extinction coefficients of 31 000, 46 000, and 59 000 $\text {L mol}^{-1}\ \text {cm}^{-1}\ \text {at}\ 10\ \text {mg/L}$ for the unfiltered, 40 nm bandpass and 20 nm bandpass configurations, respectively. The fit was performed from the entry point to a distance equal to the attenuation length.

At a concentration of just 4 mg/L, the mean absolute error (24 $\upmu \text {m}$) is more than two orders of magnitude smaller than the attenuation length (7 mm). The unexpectedly small error may be explained by the choice of reference image. For each dye concentration, a still-water image was captured and used for demodulation. Such concentration-specific reference images could partly compensate for what would otherwise be a larger systematic error, since surface elevation is measured relative to the reference plane. The geometric centroid of the subsurface emissions is, however, a function of surface topography and viewing angle, meaning that residual errors remain.

### Illustrative applications

To demonstrate the method, we present two experimental cases involving simultaneous PIV and free-surface profilometry. A further example may be found in Babiker et al. (2026).

#### Flow behind a cylinder

**Fig. 11:**
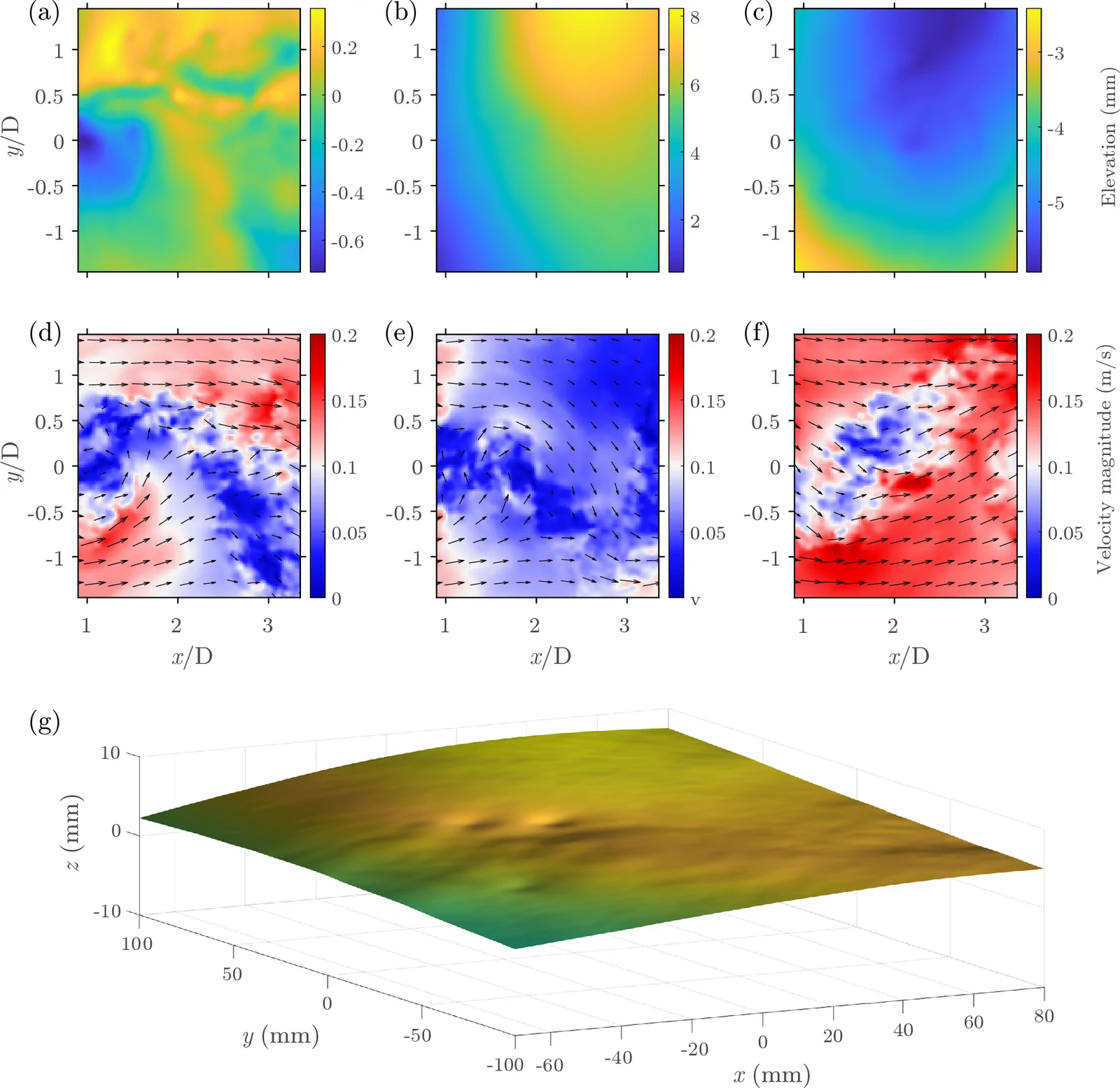
Application of the technique to flow behind a cylinder at $Re_D \approx 7000$ with waves. **a**, **b** and **c** show contours of surface elevation while **d**, **e** and **f** show contours of velocity magnitude measured 2 cm beneath the surface (vectors subsampled by a factor of 12 for clarity). Three instants in time are shown: prior to wave arrival (a and d), during the passage of a wave crest (b and e), and trough (c and f). **g**: Visualization of surface dimples induced by surface-attached vortices advecting over the wave

The first case considers the flow behind a cylinder interacting with surface waves. Despite the technological importance of cylinders affected by currents and waves, for example with respect to scour around monopiles, few fundamental studies of the fluid mechanics of the combined system exist. The relatively sparse literature largely considers the flow immediately around the cylinder for purposes of scour prediction, following the seminal work of Sumer et al. (1997), while little direct attention has been given to the lee-side wake. It has been observed, however, that a wave superposed on the current flowing past a cylinder can completely suppress the von Kármán vortex shedding (Gunnoo et al. 2016). More broadly, vortex shedding from bluff bodies has served as a canonical system for studying the coupling between surface deformation and the subsurface velocity field (Dabiri 2003; Savelsberg and van de Water 2009; Ng et al. 2010).

A vertical cylinder of diameter D=7 cm was placed in a uniform flow, piercing the free surface. The water contained fluorescein dye at a concentration of 10 mg/L. The water surface topography and the underlying flow field in a horizontal plane 2 cm beneath the quiescent water level (8 cm) were captured at a rate of 15 Hz. The mean flow velocity was U=0.1 m/s, corresponding to a Reynolds number of $Re_D=UD/\nu \approx 7000$. Following a period of steady flow, the wavemaker was activated at 1 Hz to generate waves with a wavelength of approximately 83 cm, and the interaction between the vortex street and the waves was recorded.

In Fig. 11, contours of surface elevation (top row) and velocity magnitude (middle row) are shown for three instants in time: without waves (left) and at the passing of a wave crest (middle) and trough (right). Before the waves arrive, in panels (a) and (d), the meandering wake of the cylinder is evident, along with flow separation just behind the cylinder. The fall in pressure due to separation of the boundary layer and the ensuing vortex behind the cylinder around x/D=1 generates a depression in the free surface, seen in the contours of surface elevation. After activation of the wavemaker, the waves dominate the motion of the free surface and strongly influence the velocity field. The waves, propagating against the mean current, result in a periodic increase in velocity magnitude beneath wave troughs (panel (f)) and a decrease beneath wave crests (panel (e)).

Figure 11g shows a perspective view of a wave crest, on top of which three dimples can be seen. These are imprints of surface-attached vortices shed by the cylinder and have a depth of about 0.1 mm. The ability to resolve surface perturbations which differ in scale by almost two orders of magnitude is a particular strength of projection methods such as FPP.

**Fig. 12:**
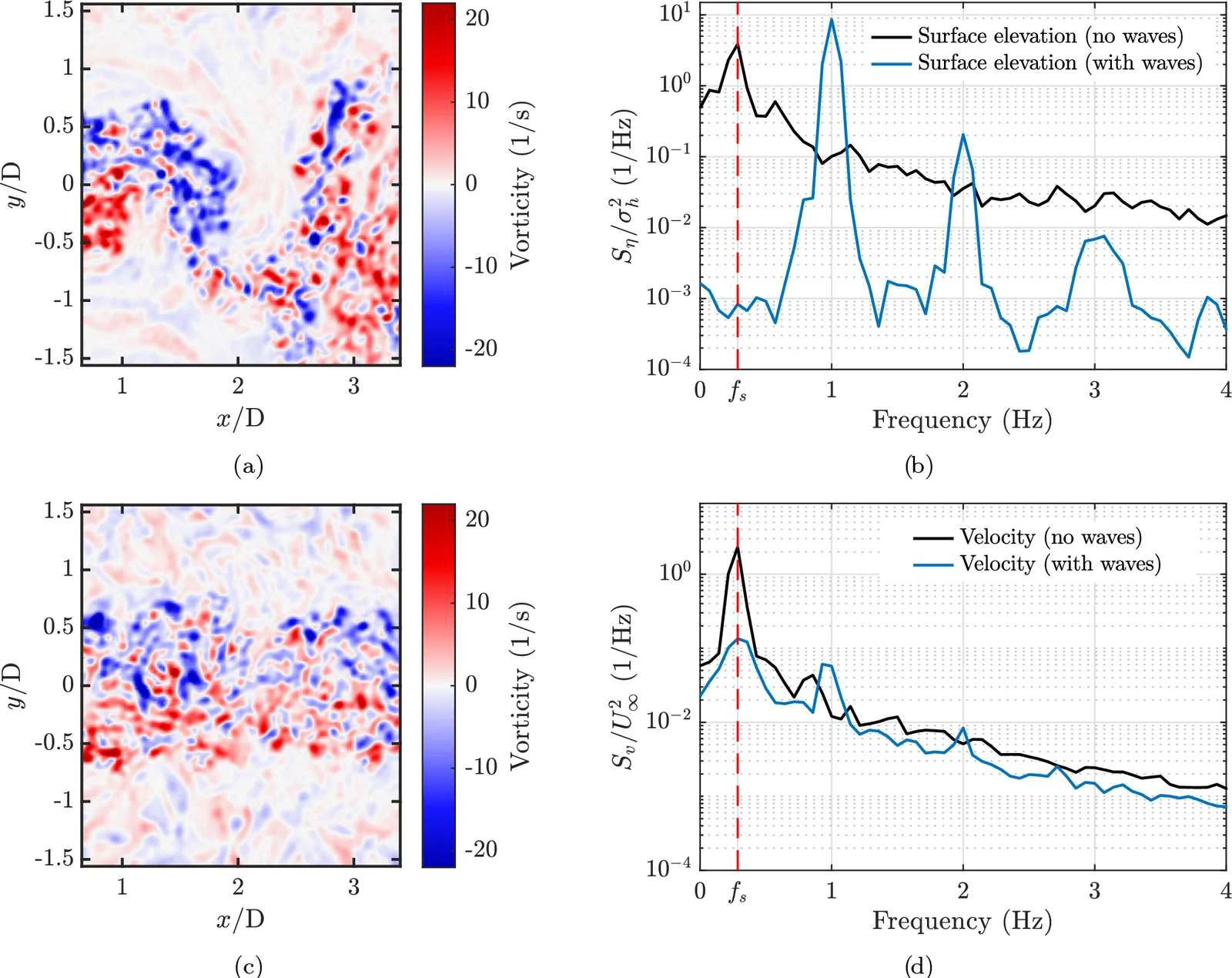
Instantaneous subsurface vertical vorticity fields behind a cylinder in turbulent flow in the absence (**a**) and presence (**c**) of counterpropagating surface waves. The corresponding power spectral densities are shown for surface elevation (**b**) and transverse velocity fluctuations (**d**), with the shedding frequency $f_s$ indicated by the dashed line

Figure 12 demonstrates the phenomenon of vortex street suppression with striking clarity. Instantaneous snapshots of the vorticity field before (Fig. 12a) and after (Fig. 12c) activation of the wavemaker reveal an almost immediate suppression of vortex shedding in the wake; an animation of this process is provided in the supplementary material (Online Resource 1).

To quantify this effect, we computed the power spectral density (PSD) of the surface elevation and the transverse velocity component, as shown in Figs. 12b and 12d, respectively. The PSD was estimated using Welch’s method (Welch 1967), applying a Hamming window to segments of 210 samples in time with 50% overlap, with a total dataset length of 600 samples. The spectra were calculated by spatially averaging the PSDs along lines of constant *y*/*D*. For the transverse velocity, the centreline (y/D=0) was used. However, because the shedding signature in the surface elevation is weak along the symmetry line, the elevation spectra were calculated using data along y/D=0.5.

The transverse velocity spectrum (Fig. 12d) exhibits a peak in energy at the shedding frequency, 0.286 Hz. This corresponds to a Strouhal number St=fD/U of 0.20, in agreement with the experiments of Roshko (1954). After activation of the wavemaker, the peak remains but is more than an order of magnitude weaker. Interestingly, a peak at 1 Hz and its harmonic at 2 Hz are conspicuous in the transverse velocity spectrum, even though the orbital velocity due to the waves alone has only a very small transverse component from imperfect wave generation and wave scattering from ambient turbulence (Smeltzer et al. 2023). The presence of these peaks indicates coupling between the wave field and the wake dynamics.

**Fig. 13:**
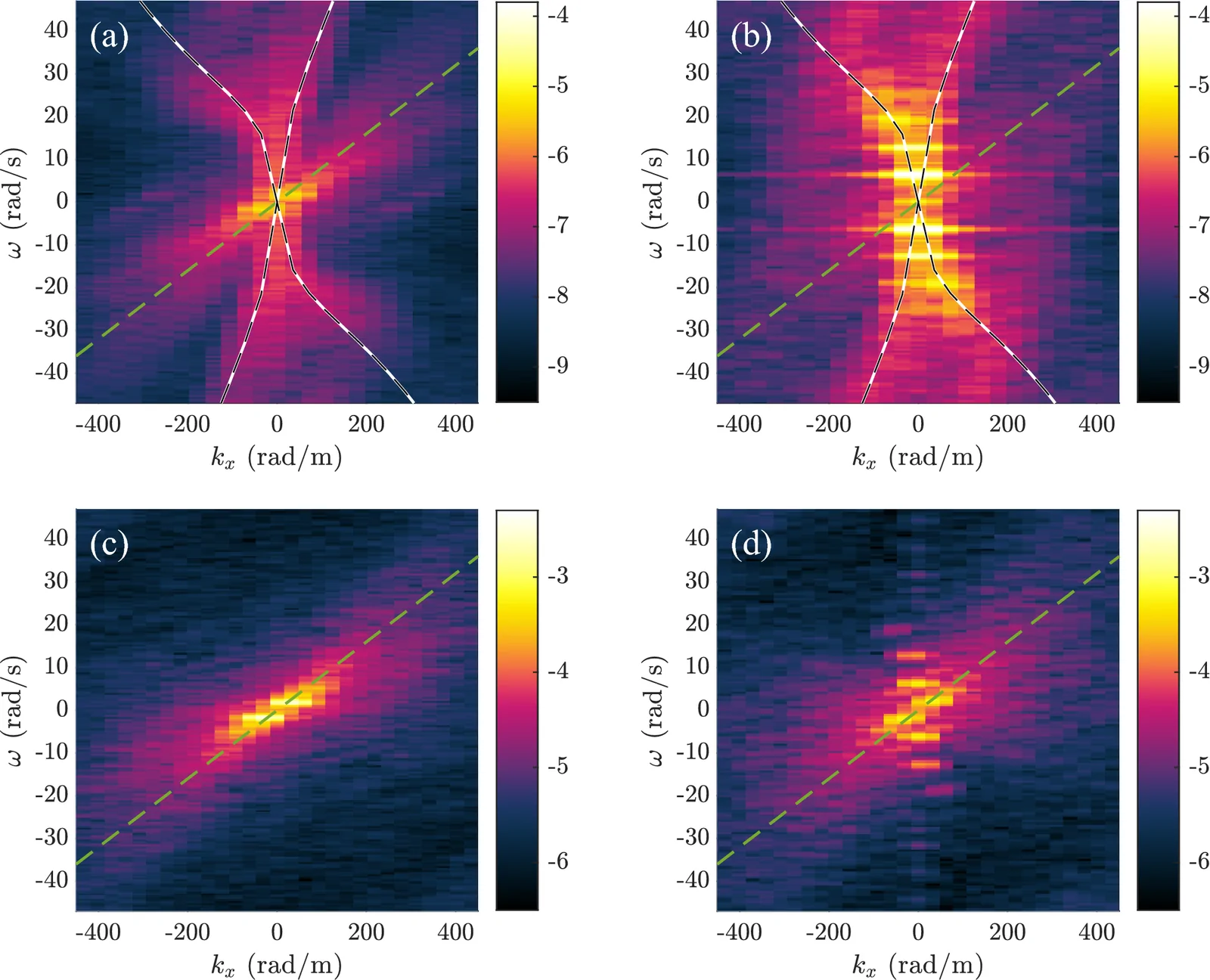
Wavenumber–frequency spectra $\log _{10} E(k,\omega )$ of the surface elevation (top row) and the transverse velocity component (bottom row). The left column shows pure vortex shedding from a cylinder (a,c), while the right column (b,d) shows vortex shedding coupled with surface waves. The black and white dashed curves show the Doppler-shifted linear dispersion relation, and the dashed green line indicates the convection line $\omega =U_\textrm{avg} k_x$, where $U_\textrm{avg} = 0.08$ m/s

The surface elevation spectrum (Fig. 12b) shows a peak at the shedding frequency (0.286 Hz) prior to wavemaker activation. After wave generation begins, the power spectrum is dominated by the waves, seen as peaks at the frequency of the wavemaker (1 Hz) and its harmonics. This is expected, given that the wave amplitude is almost two orders of magnitude larger than the oscillations resulting from vortex shedding. Notably, however, there is no longer an observable peak at the shedding frequency in the surface elevation spectrum.

Space- and time-resolved measurements allow analysis beyond the frequency spectra of Fig. 12. Figure 13 shows wavenumber–frequency spectra at $k_y=0$ (i.e. along the streamwise direction) for surface elevation (top row) and transverse velocity (bottom row). The green dashed line indicates the convection line $\omega =U_\textrm{avg} k_x$ at $U_\textrm{avg}=0.08$ m/s (see, e.g. Bullee et al. 2024), where $U_\textrm{avg}$ is the average streamwise velocity over the measurement domain. This is smaller than the free-stream velocity of 0.1 m/s due to the velocity deficit in the cylinder wake. The Doppler-shifted linear dispersion relation is overlaid as black and white dashed curves. The surface elevation spectra were computed over 0.5<y/D<1, whereas the velocity spectra were computed over the full measurement domain. Before wave generation, the surface spectrum, panel (a), shows energy distributed along both the dispersion relation (due to ambient waves) and the convection line, with distinct peaks at the shedding frequency. Velocity variations in the wake of the cylinder smear spectral energy around the convection line, as seen in both surface and velocity spectra. The transverse velocity spectrum, panel (c), also exhibits shedding peaks, with a corresponding streamwise spatial period of approximately 20 cm. After wave generation (right column), the surface spectrum, panel (b), is dominated by the wave frequency and its harmonics. The footprint of the waves is also seen in the velocity spectrum, panel (d). However, unlike the surface spectrum, the shedding frequency remains detectable, though at considerably diminished amplitude, consistent with Fig. 12d.

Gunnoo et al. (2016) remark that suppression of the von Kármán vortex shedding can occur with sufficiently large wave amplitudes, but the physical process behind this suppression remains unclear. In the present work, the interaction between waves and vortex shedding is presented primarily to demonstrate the capabilities of the measurement technique. Indications are, however, that this interaction could be fertile ground for future studies.

#### Droplet impact

**Fig. 14:**
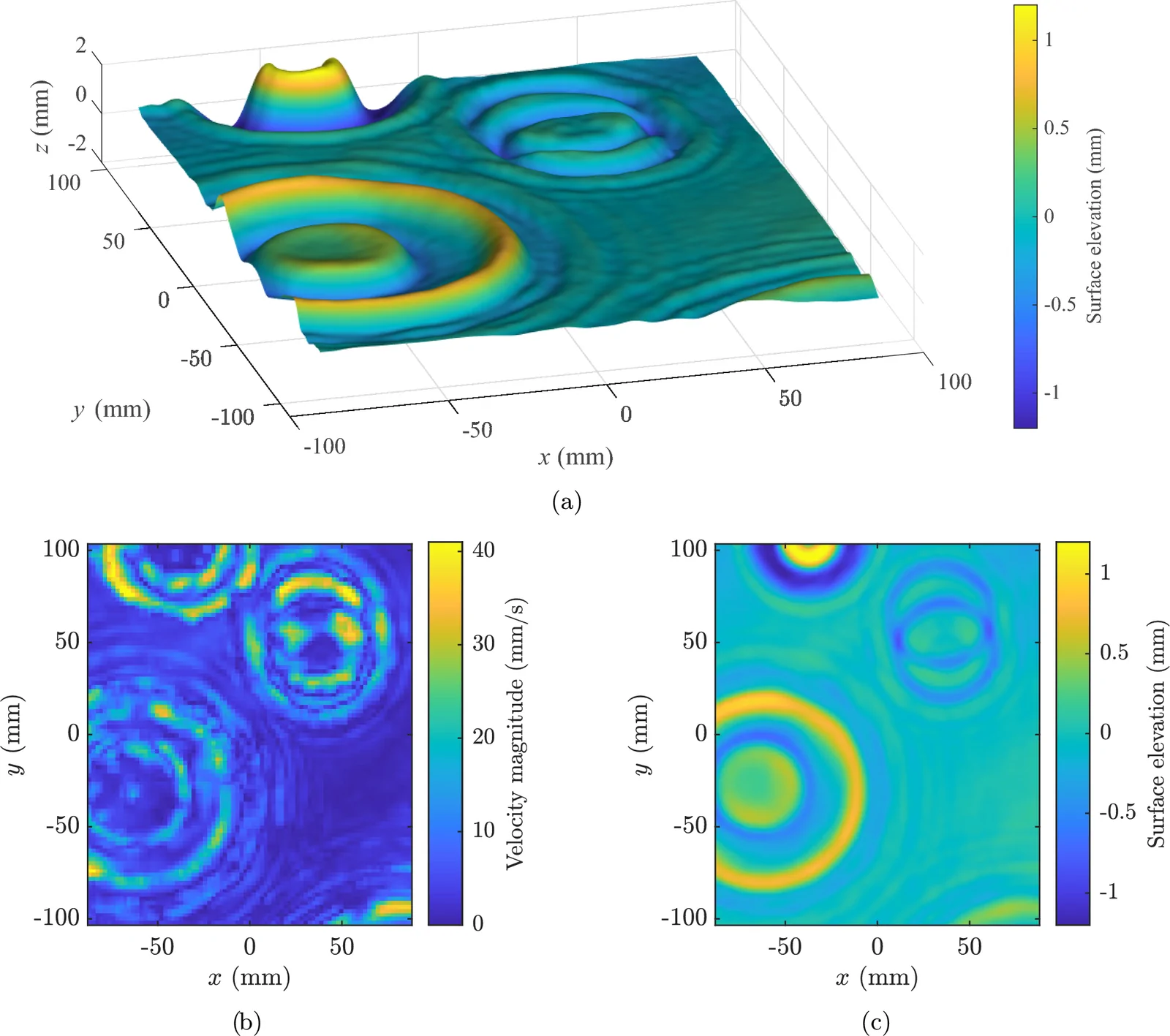
Application of the technique to droplet impacts on an initially quiescent surface. We let droplets fall randomly, and the frame shown is an arbitrarily chosen snapshot. **a** Three-dimensional reconstruction of the free surface. **b** Contours of horizontal velocity magnitude measured 1 cm below the surface. **c** Contours of surface elevation relative to the quiescent water level

**Fig. 15:**
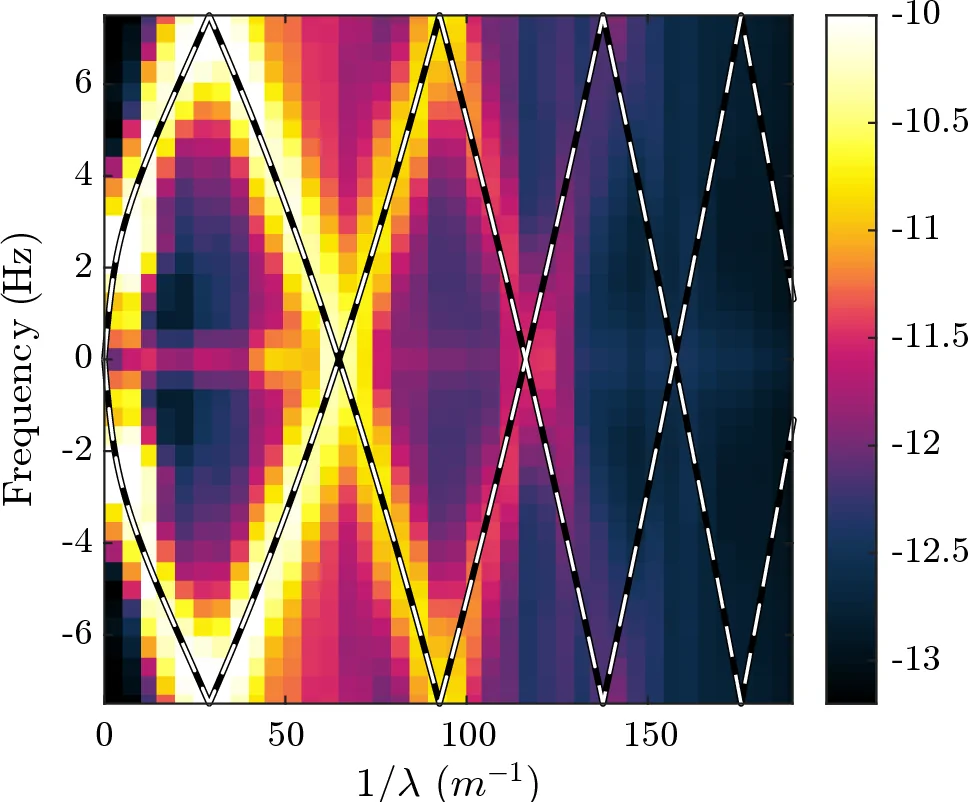
Wavenumber–frequency spectrum $\log _{10} E(k,\omega )$ for waves resulting from random droplet impacts. The curves show the linear dispersion relation, artificially aliased to facilitate comparison with experimental data

As the second demonstration case, we measure the surface deformation and subsurface velocity field during the impact of falling droplets on an initially quiescent water surface. The impact of a falling droplet on a free surface has previously been used as a benchmark for simultaneous surface and subsurface measurements (Steinmann et al. 2021). The water depth was 8 cm, the subsurface measurement plane was located 1 cm beneath the free surface, and images were recorded at 15 Hz.

Figure 14a shows a 3D reconstruction of the surface at a single instant. The interference pattern of two ring waves is apparent on the top right of the plot. Note that surface amplitudes are highly exaggerated by the different axis scaling, for visibility. Capillary waves of amplitude as low as 50 $\upmu \text {m}$ can be seen at the same time as the much larger and steeper ring waves on the top and bottom left. In Fig. 14b, contours of velocity magnitude are shown for the same instant in time, while Fig. 14c shows contours of surface elevation. While the subsurface velocity measurements exhibit some noise due to the highly three-dimensional nature of the impact flow, the spatial correspondence between the surface deformation and the flow features remains clear.

A wavenumber–frequency spectrum E(k,ω) of the surface, overlaid with the linear dispersion relation, allows us to estimate the spatial resolution limit of the profilometry. The spectrum in Fig. 15 was computed using a recording containing 600 snapshots (40 s) of surface elevation data. Due to the 15 Hz acquisition rate (limited by the laser repetition rate), the experimental spectrum is temporally aliased. To enable a direct comparison, we folded the theoretical dispersion curve to match this aliasing, resulting in the sawtooth-like appearance of the curve. The experimental energy distribution shows excellent agreement with the theoretical curve up to a wavenumber of $1/\lambda = 150\,\textrm{m}^{-1}$, corresponding to a wavelength of 6.7 mm. While it is unclear whether this limit arises from the 2.7 mm fringe spacing or the spectral noise floor, Fig. 15 confirms that capillary waves are accurately resolved down to wavelengths of 6.7 mm.

## Challenges and practical recommendations

This section outlines specific technical challenges encountered during the experimental campaign and offers practical recommendations for implementing this technique.

- Projector filter bandwidth: The attenuation length of light was less critical to measurement accuracy than initially anticipated. As a result, the 20 nm bandwidth filter proved unnecessarily restrictive. The 40 nm filter transmits more light, yielding a brighter projected pattern and a higher signal-to-noise ratio. Despite the accompanying reduction in fringe contrast from increased subsurface emissions, this brightness advantage dominates, and the 40 nm filter yielded a slightly lower MAE. When the projector input was adjusted to equalize the output brightness of both configurations, thereby removing the SNR advantage of the 40 nm filter, the 20 nm filter provided only a marginal reduction in MAE (on the order of a few micrometres).
- Projector type: Modern high-brightness commercial video projectors are typically laser based, using either three separate red, green, and blue lasers or a single blue laser with a phosphor wheel, and in either case emit comparatively little light in fluorescein’s primary absorption range (cyan, approximately 480–500 nm). One should bear in mind that the relevant quantity is the spectral overlap between emitted light and fluorescein’s absorption spectrum, not the total luminous flux (lumens). Lamp-type projectors (metal-halide or high-pressure mercury vapour, often branded “UHP” or “UHE”) on the other hand emit broad-spectrum white light that overlaps with the primary absorption band of fluorescein.
- Light source intensity: While commercial projectors offer convenience, their limited brightness prevents the use of very short exposure times. In experiments on wind-driven waves (not detailed in this manuscript), we successfully used our set-up as described, with an exposure time of 500$\upmu \text {s}$ and an acquisition frequency of 2 kHz. However, for applications requiring significantly shorter exposure times, a high-power laser-based light source (for instance similar to that of Roth et al. (2020)) could be necessary to achieve sufficient brightness. This might require a number of modifications to other parts of the set-up, beyond our present scope.
- Projector placement: Care must be taken to orient the projector so that the exhaust fan does not direct hot air across the optical path of the profilometry camera. Turbulent hot air induces refractive index fluctuations, resulting in image jitter that can easily be mistaken for surface movement.
- Resolution limit: Drawing from the experiments presented in this work, as well as additional experiments, the effective spatial resolution limit is approximately twice the fringe wavelength. To incorporate a margin of safety, we recommend selecting a fringe wavelength no larger than 0.4 times the smallest length scale of interest $L_\textrm{min}$, i.e. $\lambda _{f} \le 0.4 L_\textrm{min}$.
- Surface contaminants: Bubbles or flecks of material floating on the surface appear as bright regions in the profilometry images, causing large local perturbations in the phase field. Contaminants should therefore be minimised where possible; however, some contamination is often unavoidable, particularly in large flumes. To address this, our processing code detects anomalously large spatial gradients in the phase field and logs their locations. These flagged regions can then be masked out of the resulting surface elevation fields.

## Conclusions

We have demonstrated a new method for simultaneous measurement of a moving free water surface and the subsurface velocity field, using a combination of Fringe Projection Profilometry (FPP) and particle image velocimetry (PIV). The method involves dyeing the water with a fluorescent dye (fluorescein) which effectively makes the water opaque to some wavelengths of light and transparent to others. Fluorescein strongly absorbs blue wavelengths and emits in the green.

A cyan sine-wave pattern is projected onto the surface by a video projector; the dye’s strong absorption of this light allows the surface elevation to be deduced from the displacement of the observed pattern. A PIV light sheet is created with a green laser of wavelength 532 nm, where fluorescein has weak, but nonzero, absorption.

A combination of optical filters allows for clean images for both PIV and profilometry. A long-pass filter was applied to the FPP camera to reject specular reflections (which are cyan) and retain only the fluorescence (which is green). Some of the green laser light was absorbed and reemitted, causing noise in the PIV images; we effectively removed this with a narrowband filter centred at the laser’s wavelength.

The surface elevation measurements were validated against single-point Laser-Induced Fluorescence (LIF) measurements. We found a mean absolute error (MAE) of 17 $\upmu \text {m}$ at the highest fluorescein concentration, 25 mg/L. As expected, errors increase at lower concentrations as the attenuation length of the projected light increases. However, they do so surprisingly slowly; even at a concentration of 4 mg/L the 75th percentile of the error remains below 60 $\upmu \text {m}$ and the MAE is 24 $\upmu \text {m}$, sufficient for many purposes. Beyond approximately 12 mg/L, where the MAE is 18 $\upmu \text {m}$, increasing the concentration of fluorescein did not result in an appreciable improvement in accuracy. In terms of PIV performance, the correlation values for our set-up were essentially unchanged up to concentrations of 8 mg/L. Beyond this point, correlation values declined but remained robust (>0.6 at 20 mg/L) due to the effective noise suppression of the narrowband filter.

The capabilities of the method were demonstrated through two experimental cases. First, we measured flow past a vertical cylinder at a Reynolds number of $Re_D = UD/\nu \approx 7000$. We observed that the addition of surface waves could suppress the von Kármán vortex street behind the cylinder. Notably, the system was able to resolve small dimples from surface-attached ‘bathtub’ vortices, 0.1 mm deep atop waves of centimetre amplitude, demonstrating the ability of FPP to measure accurately across disparate scales. As a second example, we captured the simultaneous surface ring waves and subsurface velocity fields produced by droplet impacts. The technique therefore proves to be both accurate and practical, offering a versatile solution for a wide range of fluid dynamics applications.

## Supplementary Information

Below is the link to the electronic supplementary material.Supplementary file 1 (mp4 16302 KB)

## Data Availability

The data supporting the findings of this study are available for download from the Norwegian national data repository DataverseNO at https://doi.org/10.18710/MWEHEM.

## Declarations

### Code availability

The code used to generate the surface elevation data presented in this study is publicly available on GitHub (https://doi.org/https://github.com/asemati/FTProfilometry).
